# The SCR-17 and SCR-18 glycans in human complement factor H enhance its regulatory function

**DOI:** 10.1016/j.jbc.2024.107624

**Published:** 2024-08-02

**Authors:** Xin Gao, Hina Iqbal, Ding-Quan Yu, Jayesh Gor, Alun R. Coker, Stephen J. Perkins

**Affiliations:** 1 Division of Biosciences, Department of Structural and Molecular Biology, University College London, London, UK; 2 Division of Medicine, University College London, London, UK

**Keywords:** analytical ultracentrifugation, atomistic modeling, complement, molecular dynamics, small angle X-ray scattering, surface plasmon resonance

## Abstract

Human complement factor H (CFH) plays a central role in regulating activated C3b to protect host cells. CFH contain 20 short complement regulator (SCR) domains and eight N-glycosylation sites. The N-terminal SCR domains mediate C3b degradation while the C-terminal CFH domains bind to host cell surfaces to protect these. Our earlier study of *Pichia*-generated CFH fragments indicated a self-association site at SCR-17/18 that comprises a dimerization site for human factor H. Two N-linked glycans are located on SCR-17 and SCR-18. Here, when we expressed SCR-17/18 without glycans in an *Escherichia coli* system, analytical ultracentrifugation showed that no dimers were now formed. To investigate this novel finding, full-length CFH and its C-terminal fragments were purified from human plasma and *Pichia pastoris* respectively, and their glycans were enzymatically removed using PNGase F. Using size-exclusion chromatography, mass spectrometry, and analytical ultracentrifugation, SCR-17/18 from *Pichia* showed notably less dimer formation without its glycans, confirming that the glycans are necessary for the formation of SCR-17/18 dimers. By surface plasmon resonance, affinity analyses interaction showed decreased binding of deglycosylated full-length CFH to immobilized C3b, showing that CFH glycosylation enhances the key CFH regulation of C3b. We conclude that our study revealed a significant new aspect of CFH regulation based on its glycosylation and its resulting dimerization.

The complement system, an integral part of the human innate immune response, functions as an enzymatic cascade designed to eliminate damaged cells or potential pathogens before they can establish an infection. The alternative pathway of activation involves the transformation of inactive C3 through a “tickover” mechanism into a form known as C3u or C3_H2O_. C3u closely resembles the activated form of C3 termed C3b. C3u enables the cleavage of C3 through the C3 convertase, thereby generating active C3b which attaches itself to the surfaces of compromised cells, marking them for immune-mediated elimination. Complement factor H (CFH), a key regulatory protein, safeguards host cells from this complement-mediated destruction by interacting with surface-bound C3b through its C-terminal region on anionic cell surfaces (1, 2). The subsequent binding of CFH to complement factor I deactivates C3b by converting it into inert iC3b (3).

CFH is a glycoprotein of size about 154 kDa and comprised of 20 short complement regulator (SCR) domains. Each SCR domain contains around 61 amino acids and joined to their neighbors by linkers of three to eight amino acids (Fig. 1*A*) (4). There are nine N-linked glycosylation sites, of which eight are occupied by biantennary complex-type glycans (5). Due to its size, glycosylation, interdomain flexibility, and self-association tendencies, determining the complete molecular structure of CFH has been challenging. Nonetheless, to date, high-resolution molecular structures for 12 SCR domains have been elucidated by X-ray crystallography and for seven SCR domains by NMR spectroscopy (6). Currently, only SCR-9, SCR-14, and SCR-17 are without high-resolution structures, and their molecular models have been developed using homology modeling (Fig. 1*A*). Electron microscopy and small angle scattering have shown that CFH adopts a folded-back SCR domain structure (6, 7, 8). The C-terminal SCR-19 and SCR-20 domains interact with C3b and its thioester domain C3d as well as with polyanionic saccharides found at host cell surfaces (3, 9).

**Figure 1:**
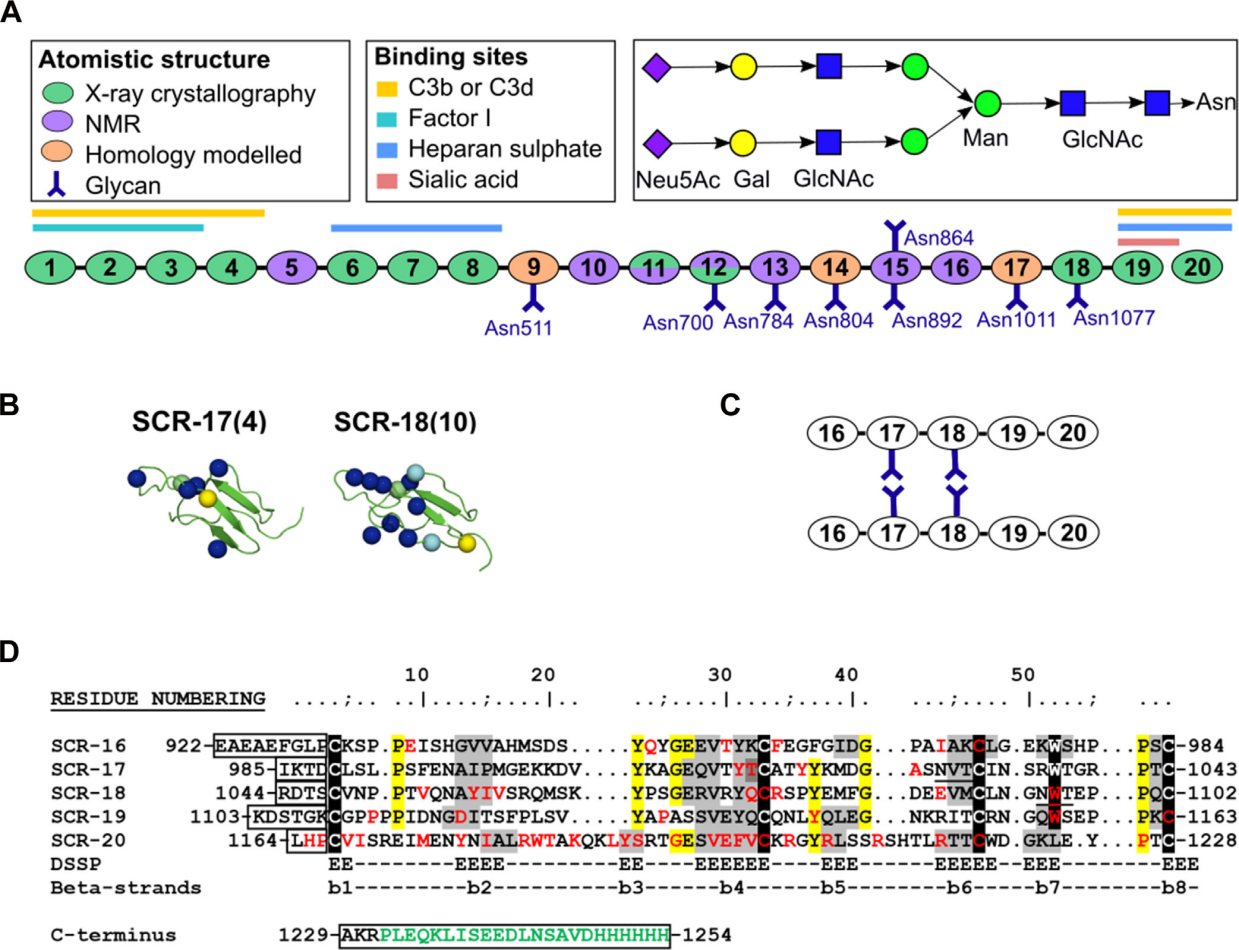
**Domain structures and sequences for the Factor H C-terminal domains**. *A*, schematic diagram of the 20 SCR domains in Factor H, showing their functional significance, the location of eight N-linked glycosylation sites, and the structure of a typical WT N-linked glycan. The Y-symbol represents an N-linked glycan. *B*, schematic view of the SCR-17 and SCR-18 protein structures with their disease-associated mutations shown as *blue spheres* (aHUS), *green spheres* (aHUS and C3G), and *cyan spheres* (C3G). The Asn1011 and Asn1077 glycosylation sites in SCR-17 and SCR-18 are shown as *yellow spheres*. The number of mutations in each domain is bracketed beside the domain label. *C*, a schematic diagram of a putative SCR-16/20 dimer formed by the side-by-side association of SCR-17 and SCR-18. *D*, the five C-terminal domain sequences are shown, with the five conserved Trp and Cys residues highlighted in *black* and other conserved residues in *yellow*. The two glycosylation sites at positions 44 and 51 are underlined. The inter-SCR linkers are boxed at the *left*. Residues highlighted in *gray* have β-strand secondary structures. Disease-associated residues are colored in *red*. If expressed with a hexa-His tag, the C-terminal sequence in *green* will be present. The N-terminal sequence EAEAEF is the α-factor signal and the EcoRI site. Artwork adapted from (17). aHUS, atypical hemolytic uremic syndrome; C3G, C3 glomerulopathy; SCR, short complement regulator.

Atypical hemolytic uremic syndrome (aHUS) is an infrequent condition defined by renal endothelial cell damage due to disrupted complement regulation. It frequently results in end-stage kidney failure (10). There is a strong link between aHUS and genetic variants in CFH (11, 12, 13). These CFH variants cause a loss of regulatory function, undermining the protection of renal endothelial surfaces and instigating complement activation on these surfaces (14). In the most recent review of CFH variants, 190 disease-related variants were identified (13, 15), many being located within the five C-terminal SCR domains of CFH. The online database (https://www.complement-db.org/) lists six variants in SCR-16, four in SCR-17, ten in SCR-18, seven in SCR-19, and 37 in SCR-20 (Fig. 1*D*). The most variants occur in SCR-20, which contains the binding sites for C3b, C3d, and polyanions and affecting the ability of CFH to recognize and safeguard host cells. Other renal conditions linked to complement include C3 glomerulopathy, which affects the N terminus of CFH (13).

Full-length CFH forms 4 to 15% dimers at normal CFH serum concentrations of 0.8 to 3.6 μM (0.116–0.562 mg/ml) (4, 16). Analytical ultracentrifugation (AUC) showed that the single SCR-17H and SCR-18H domains each exhibited significant dimer formation. These observations suggest that the dimer is formed through the SCR-17/18 domains (Fig. 1*C*) (17). Such a dimer site would provide further protection to the host surface in inflammatory conditions characterized by excessive C3b deposition and high local concentrations of CFH. Two N-linked glycosylation sites were located on SCR-17/18, which may contribute to its dimerization. In order to investigate the C-terminal dimerization of CFH and its significance, here we have performed AUC and mass spectrometry studies of two SCR-17/18 forms with and without glycans. By AUC and small-angle X-ray scattering (SAXS), we have characterized the solution structures of these different forms and used atomistic modeling simulations of the SAXS data sets to characterize dimer formation (18, 19). Finally, to establish the functional significance of CFH C-terminal glycosylation, surface plasmon resonance (SPR) was utilized to study its effect on the ligand-binding affinities of CFH. Our combined data sets show that the CFH glycans play a significant role in the conformation and perform enhanced functionality of CFH binding to host cell surfaces.

## Results

### Purification and characterization of deglycosylated SCR-17/18 of CFH

**Figure 2:**
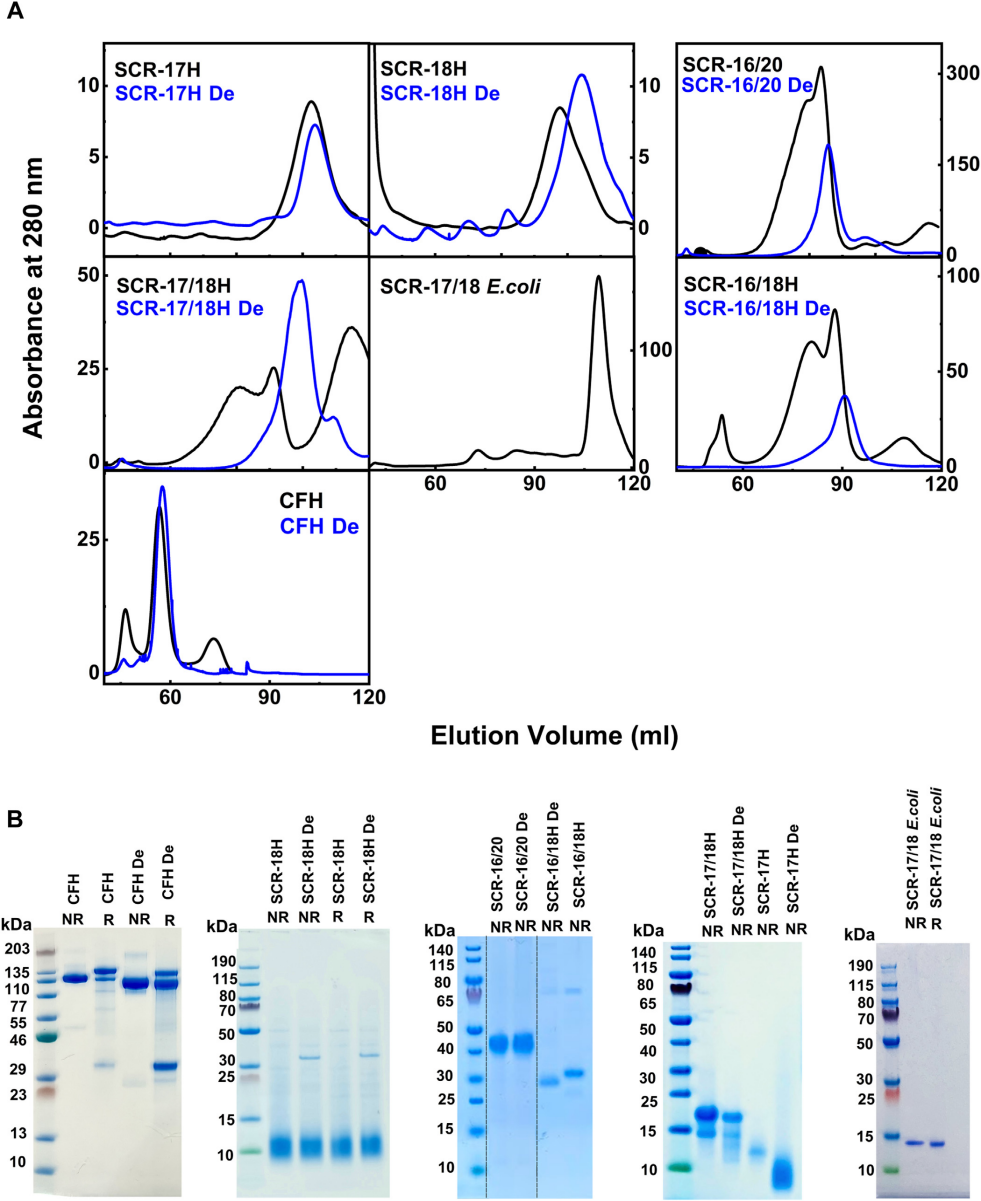
**Purification and characterization of five SCR fragments and CFH before and after deglycosylation**. The suffix De denotes deglycosylation with PNGase F. *A*, size-exclusion chromatography elution profiles of the five SCR fragments and CFH in Hepes EDTA-free buffer. The deglycosylated SCR profiles are in *blue*. *B*, SDS-PAGE of the purified SCR fragments and CFH. In the *left*-most or *right*-most lanes, the molecular mass markers are denoted in kDa. The other lanes correspond to nonreduced (NR) and reduced (R) proteins. The SDS-PAGE for SCR-16/18H and SCR-16/20 were cut from a single gel that is included in Appendix S1, and the splice borders are denoted by *vertical dashed lines*. CFH, complement factor H; SCR, short complement regulator.

**Figure 3:**
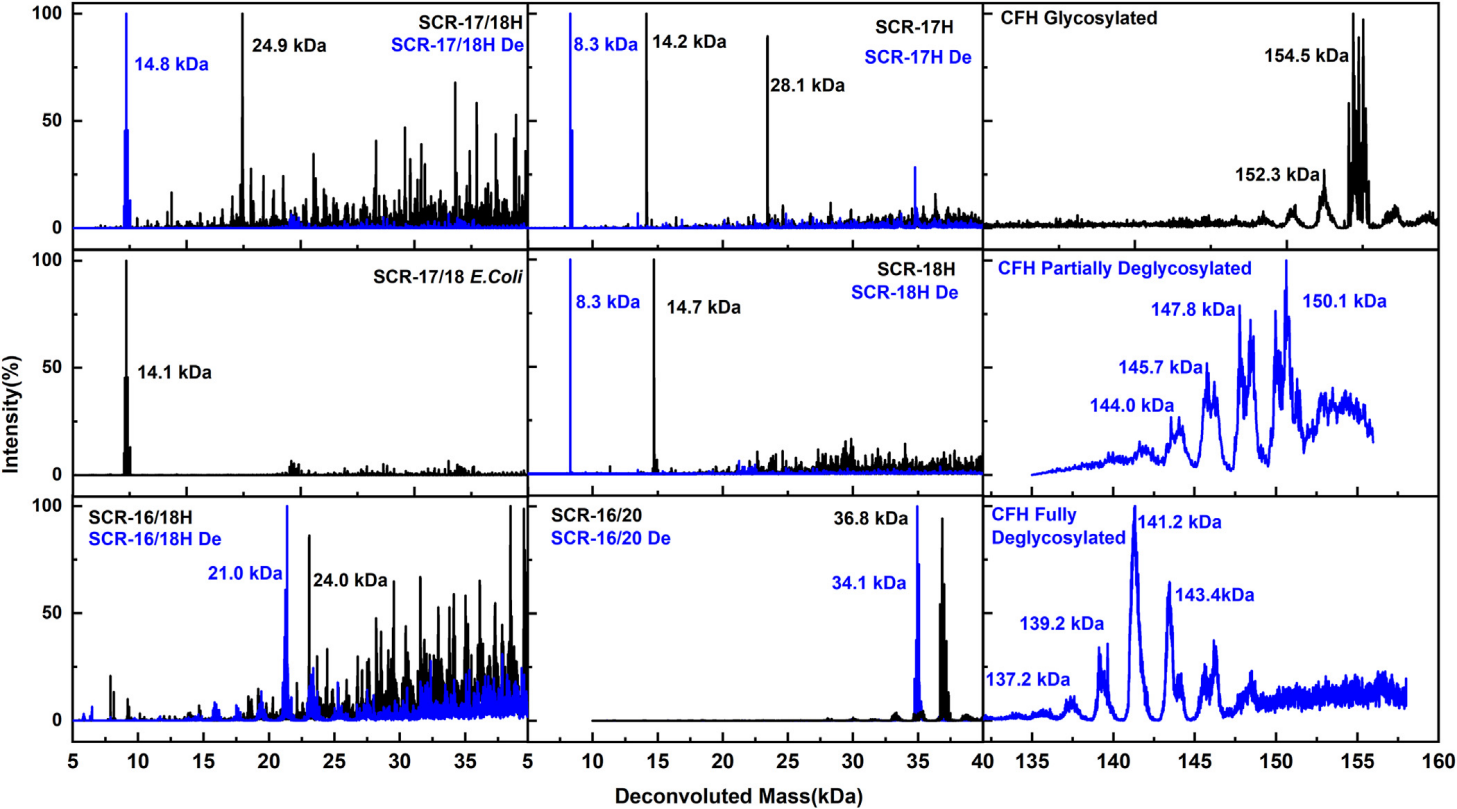
**Mass spectrometry of the SCR fragments and CFH.** The molecular mass values are shown near each major peak. Glycosylated proteins are shown in *black* and the PNGase F-deglycosylated proteins are shown in *blue*. CFH, complement factor H; SCR, short complement regulator.

**Figure 4:**
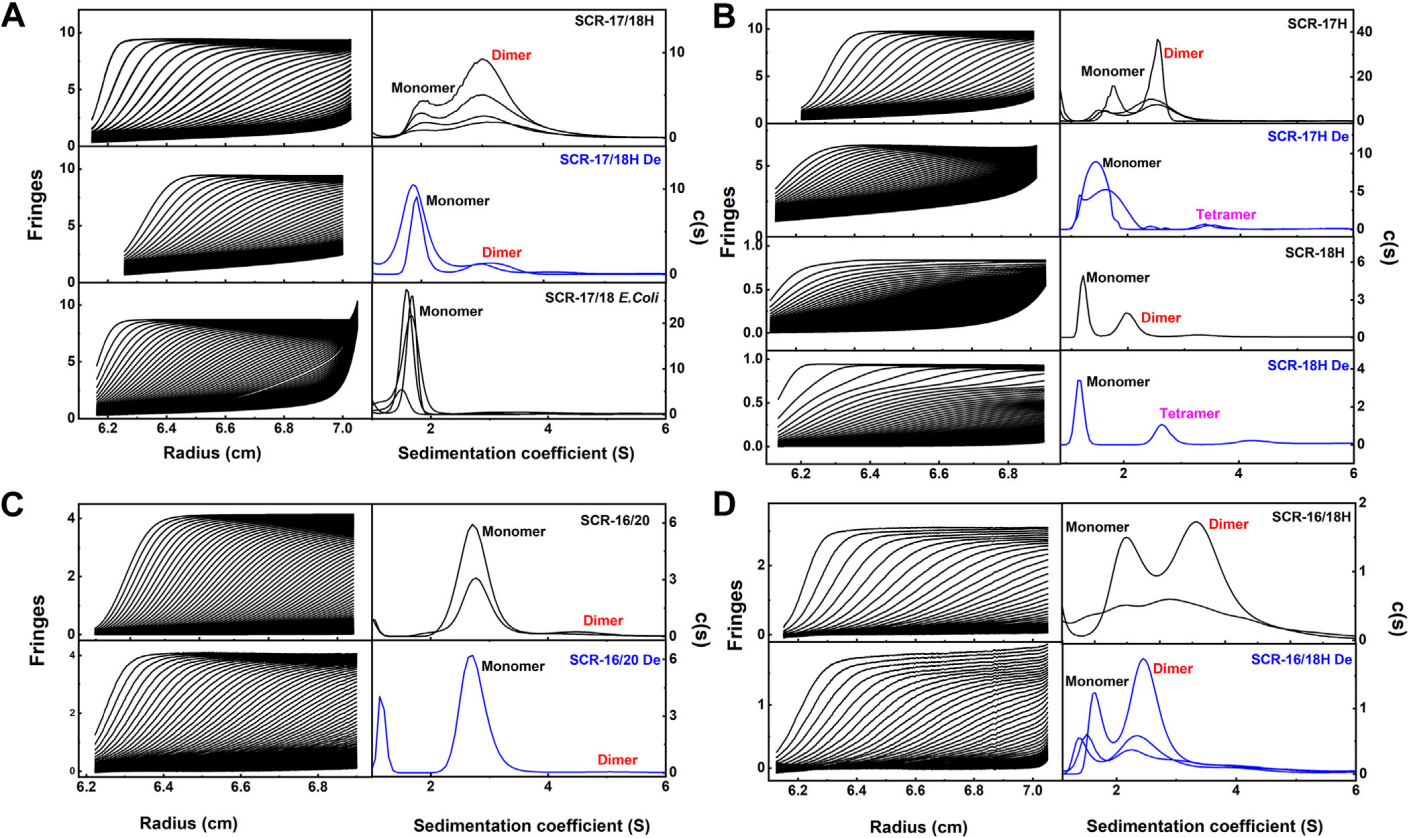
**Sedimentation velocity analyses of five SCR fragments.** The SEDFIT boundary fits are shown as *black lines* (*left panels*), and the size-distribution analyses *c(s)* are shown in the *right* panels. All samples were measured using Hepes buffer. *A*, the two-domain SCR-17/18 fragments was studied in three forms. SCR-17/18H at 2.0 mg/ml, 1.5 mg/ml, 1.0 mg/ml, and 0.5 mg/ml; SCR-17/18H De at 2.0 mg/ml and 1.0 mg/ml; SCR-17/18H *Escherichia coli* in 2.4 mg/ml, 2.0 mg/ml, 1.5 mg/ml, and 0.5 mg/ml. The monomer peaks for SCR-17/18H, SCR-17/18H De, and SCR-17/18 *E. coli* in the *c(s)* analyses corresponds to *s*_*20,w*_ values of 2.4 to 2.5 S, 2.2 to 2.3 S, and 1.8 to 1.9 S, respectively, while dimer peaks for SCR-17/18H and SCR-17/18H De were seen at 3.8 to 3.9 S and 3.6 to 3.7 S, respectively. No dimer peak was observed for SCR-17/18 *E. coli*. *B*, the single-domain SCR-17 and SCR-18 fragments were studied as follows: SCR-17H at 1.0 mg/ml, 0.5 mg/ml, 0.4 mg/ml; SCR-17H De at 0.5 mg/ml and 0.25 mg/ml; SCR-18H at 0.5 mg/ml; and SCR-18H De at 0.5 mg/ml. The monomer peak in the *c(s)* analyses corresponds to *s*_*20,w*_ values of 1.85 to 1.95 S, 1.70 to 1.80 S, 1.60 to 1.70 S, and 1.50 to 1.60 S for SCR-17H, SCR-17H De, SCR-18H, and SCR-18H De, respectively. The trace dimer peak was visible at 2.80 to 2.90 S and 2.0 S for SCR-17H and SCR-18H, respectively. No dimer peaks were observed for deglycosylated SCR-17H and SCR-18H; however, a small peak at higher *s*_*20,w*_ values was attributed to likely tetramer formation. *C*, the five-domain SCR-16/20 fragment was studied at 0.9 mg/ml and 0.5 mg/ml and that for SCR-16/20 De at 0.9 mg/ml. The monomer peak in the *c(s)* analyses corresponds to *s*_*20,w*_ values of 2.70 to 2.80 S and 2.60 to 2.70 S for SCR-16/20 and SCR-16/20 De, respectively. The very small dimer peak was visible at 4.70 to 4.80 S and 5.10 to 5.20 S, respectively before and after deglycosylation. *D*, the three-domain SCR-16/18H fragment was studied at 0.4 mg/ml and 0.1 mg/ml and that for SCR-16/18H De at 0.4 mg/ml, 0.2 mg/ml, and 0.1 mg/ml. The monomer peak in the *c(s)* analyses corresponds to *s*_*20,w*_ values of 2.20 to 2.30 S and 1.6 to 1.7 S for SCR-16/18H and SCR-16/18H De, respectively. The dimer peak was visible at 3.80 to 3.90 S and 2.80 to 2.90 S, respectively, before and after deglycosylation. SCR, short complement regulator.

Given the earlier identification of a significant dimerization site in *Pichia*-expressed SCR-17/18 (17), initially we attempted to crystallize this in order to identify the molecular basis of CFH dimerization. As the two glycans on SCR-17 and SCR-18 were expected to impede the growth of crystals, we chose to express SCR-17/18 in *Escherichia coli* so that no glycans would be present. SCR-17/18 was expressed in quantity and eluted as a sharp band by size-exclusion chromatography (Fig. 2*A*). A single well-resolved band at 17 kDa was seen by reduced SDS-PAGE (Fig. 2*B*), close to the expected value of 14.1 kDa calculated from the SCR-17/18 sequence. Mass spectrometry gave a mass of 14.1 kDa as a single peak, showing that this was pure and close to the sequence-calculated value of 14.1 kDa (Fig. 3). When this protein was subjected to sedimentation velocity AUC, much to our surprise, SCR-17/18 was clearly monomeric with a sedimentation coefficient *s*_*20,w*_ of 1.8 S (Fig. 4*A*) that was close to that of 1.64 S for monomeric SCR-19/20 (17). As the only difference between the *Pichia*-expressed and *E. coli*–expressed SCR-17/18 proteins was the presence and absence of the two N-glycans respectively, it was concluded that the glycans of SCR-17/18 were responsible for the C-terminal dimerization of CFH.

### Characterization of glycosylated and deglycosylated SCR fragments

To establish whether or not the SCR-17/18 glycans were responsible for CFH dimerization, five SCR fragments containing SCR-17/18 from our previous study (constructs SCR-16/20, SCR-16/18H, SCR-17/18H, SCR-17H, and SCR-18H) were purified using *Pichia* expression (17) and were enzymatically deglycosylated using peptide:N-glycosidase F (PNGase F) (Experimental procedures). For *Pichia* SCR-17/18, tests were made with digests that each lasted for 12 h, 18 h, and 24 h. No significant difference was seen between these times, thus 12 h was used as the final digest time. The same result was obtained for *Pichia*-expressed SCR-17, SCR-18, SCR-16/18, and SCR-16/20. Gel filtration and SDS-PAGE initially examined the integrity of the five SCR fragments (Fig. 2, *A* and *B*). SCR-17H with and without glycans eluted as a single symmetrical peak at 102.7 ml and 103.7 ml, respectively. SCR-18H with and without glycans similarly eluted as a single symmetrical peak at 97.7 ml and 104.4 ml, respectively. Deglycosylated SCR-17H and SCR-18H eluted slightly later, which indicated a reduced molecular mass compared to native SCR-17H and SCR-18H (Fig. 2*A*). For SCR-17/18H, two peaks were observed at 80.7 ml and 91.1 ml that indicated two dimer and monomer forms. Deglycosylated SCR-17/18H however showed a single peak at 99.0 ml, indicating a reduced molecular mass and a monomer form. SCR-16/20 and SCR-16/18H showed similar patterns in their elution profiles. SCR-16/20 exhibited a shoulder peak at 79.3 ml and a main peak at 83.6 ml, in distinction to deglycosylated SCR-16/20, which only exhibited a single symmetrical peak at 85.9 ml. SCR-16/18H displayed two peaks at 80.4 ml and 88.5 ml, which became a single symmetrical peak at 90.7 ml after deglycosylation. Overall, all the SCR fragments with glycans exhibited broader elution profiles. After deglycosylation, the profiles were reduced in width. The observation of single peaks showed that the oligomerized proteins were much diminished and that the glycans did influence dimer formation.

The glycosylated and deglycosylated SCR samples from gel filtration were loaded onto a 4 to 12% Bis Tris NuPAGE SDS-PAGE gel, with and without a reducing agent (Fig. 2*B*). The SCR fragments displayed *Pichia pastoris* hyperglycosylated major oligosaccharides. These corresponded to a Man_8–18_GlcNAc_2_ core with their masses between 2 to 10 kDa (20, 21). This was observable on the SDS-PAGE, where the glycosylated SCR fragments showed multiple or broader bands (Fig. 2*B*), although the results of deglycosylation were not immediately accurate.

Liquid chromatography mass spectrometry clarified the N-linked deglycosylation of the SCR fragments (Fig. 3). The SCR fragments purified from *P. pastoris* exhibited hyperglycosylation. The glycosylated native SCR-17/18H displayed a strong peak with a molecular mass of 24.9 kDa, with multiple low-intensity peaks indicating glycan mass variance and oligomerization. After its deglycosylation, these low-intensity peaks disappeared, leaving only one strong peak at 14.8 kDa, that was a good match for the expected value of 15.8 kDa for monomer SCR-17/18H without glycans. The mass of 24.9 kDa for native SCR-17/18H suggested that SCR-17/18H mostly formed dimers. The *E. coli*–expressed SCR-17/18 showed only a single peak at 14.1 kDa. Next, deglycosylated SCR-17H exhibited a molecular mass at 8.3 kDa, also closely matching the expected value of 9.6 kDa for monomer SCR-17H. Glycosylated SCR-17H displayed one strong peak at 14.2 kDa, which was almost half the size of the 28.1 kDa peak to indicate the formation of SCR-17H monomers and dimers. Glycosylated SCR-18H showed a similar peak at 14.7 kDa to SCR-17H; after deglycosylation, the molecular mass was reduced to 8.3 kDa, which was well-matched to the estimated 9.5 kDa of monomer SCR-18H without glycan. The deglycosylation of SCR-16/18H showed a molecular mass decrease from 24.0 kDa to 21.0 kDa, closely aligning with the expected mass of monomer SCR-16/18H at 22.0 kDa. SCR-16/20 showed a similar molecular weight decrease after deglycosylation, from 36.8 kDa to 34.1 kDa, the latter being a close match to the expected value of 34.4 kDa. Overall, all the SCR fragments with glycans showed lower masses after deglycosylation.

The hyperglycosylation resulting from the *P. pastoris* expression system (noted above) gave additional masses that ranged from 2 to 10 kDa. After the deglycosylations, the changes in the N-linked glycan masses ranged from 2.85 kDa to 5.7 kDa. Consequently, a high-mannose N-linked glycan, Man_23_GlcNAc_2_, with a molecular mass of 4.122 kDa was taken as the structure for the atomic build of all SCR-fragments with glycans in the simulations below.

### Characterization of glycosylated and deglycosylated CFH

Similar experiments were performed on native glycosylated CFH. The CFH and CFH-De gel filtration elution profiles showed changes after deglycosylation, which can be attributed to their lower molecular weights and reduced levels of oligomerization (Fig. 2*A*). The elution peak for CFH-De after deglycosylation increased slightly to 57.9 ml, compared to that for native CFH at 56.7 ml. SDS-PAGE likewise displayed the anticipated results (Fig. 2*B*). Nonreduced CFH exhibited a single band between 115 and 190 kDa, which agrees with the expected mass of 154 kDa calculated from the CFH sequence. Reduced CFH displayed multiple bands, with the main band again seen at 115 to 190 kDa attributed to the unreduced protein. In addition, a faint band at 30 to 50 kDa corresponds to an internal cleavage in the CFH domains. Deglycosylated CFH-De with and without reducing agents displayed bands below those of glycosylated CFH. This suggests a reduction in molecular weight following deglycosylation.

The mass spectra of glycosylated CFH displayed a strong signal with a mass of 154.5 kDa for its eight glycan chains and a smaller signal at 152.3 kDa that may correspond to the loss of a single glycan chain. This loss of 2.2 kDa corresponds well to the molecular mass of N-linked glycans with a composition of Man_3_GlcNAc_6_NeuAc_2_ (mass 2286 Da) (Fig. 1*A*). This outcome confirmed its high purity. Interestingly, the deglycosylation rate of CFH is noticeably slow. After 100 h of PNGase F cleavage, four intensity peaks with similar molecular weight decreases between peaks were observed that suggested CFH forms with three to six glycan chains were present (Fig. 3). Two glycan chains were removed comparatively quickly. Below, this is denoted as partially deglycosylated CFH. After 200 h of PNGase F cleavage, the final molecular masses for deglycosylated CFH were observed to be 137.2 kDa, 139.2 kDa, 141.2 kDa, and 143.4 kDa, indicating the removal of eight, seven, six, or five N-linked glycans to leave behind none, one, two, or three glycans on CFH in that order (Fig. 3). Below, this is denoted as fully deglycosylated CFH.

### AUC of five glycosylated and deglycosylated SCR fragments

AUC provides quantitative analyses of macromolecules to determine their masses and solution structures (22). Here, the five SCR fragments before and after deglycosylation were investigated in order to assess whether their glycans affect their dimerization (Fig. 4). Totals of 300 to 650 scans were measured by sedimentation velocity, from which around 35 interference boundaries were fitted using SEDFIT (left panels, Fig. 4). Excellent visual agreements between the fitted lines (black line) and experimental scans (circles) were obtained. The residuals (root mean square difference) were low at 0.08 to 0.015 fringes. Peaks in the size distribution *c(s)* analysis (right panels, Fig. 4) represent different molecular species, and the relative proportions of monomer and dimer can be determined from peak integrations.

For SCR-17/18H from 0.4 to 2.0 mg/ml, its glycosylated form displayed two clear *c(s)* peaks, signifying the presence of monomer and dimer (Fig. 4*A*). The *s*_*20,w*_ values were 2.45 ± 0.01 S for the monomer and 3.81 ± 0.1 S for the dimer. Their molecular masses were computed to be 23.9 ± 1.1 kDa for the monomer and 44.5 ± 2.6 kDa for the dimer, which agree well with the sequence-calculated values of 22 kDa and 44 kDa, respectively. Following deglycosylation with PNGase F, SCR-17/18H De was observed as both monomer and dimer as two separate peaks at 1.5 mg/ml and 2.0 mg/ml. Interestingly, the amount of dimer was significantly diminished. The *s*_*20,w*_ values for the monomer and dimer were unchanged at 2.27 ± 0.03 S and 3.69 ± 0.01 S, respectively. The molecular masses were 20.1 ± 0.4 kDa and 40.5 ± 1.6 kDa for the dimer, which agree well with the expected masses of 19 kDa for the monomer and 38 kDa for the dimer. There was a large increase in the monomer post-deglycosylation, from 28% to 84%, while the dimer decreased from 72% to 16%. This outcome shows that the glycans facilitate dimer formation.

For SCR-17H, the *c(s)* distribution demonstrated two peaks with *s*_*20,w*_ values of 1.90 ± 0.20 S and 2.93 ± 0.04 S (Fig. 4*B*). The *c(s)* plot gave 33% monomer and 67% dimer, yielding molecular masses of 14.9 ± 1.1 kDa and 28.6 ± 2.5 kDa for the first and second peaks, respectively. Following deglycosylation, only the monomer peak was observed with an *s*_*20,w*_ value of 1.75 ± 0.26 and 96% monomer, with no dimer but a slight tetramer. The monomer mass of 8.8 ± 1.9 kDa agreed with the mass spectrometry (Fig. 3). SCR-18H likewise exhibited monomer and dimer peaks at 1.66 S and 3.82 S, respectively. After deglycosylation, the monomer peak, with an *s*_*20,w*_ value of 1.56 S and a mass of 8.33 kDa, was present with 92% abundance and corresponded to the mass spectrometry value. The AUC data for both SCR-17H and SCR-18H indicate a large reduction in dimer formation following deglycosylation. Traces of a third peak (denoted as tetramer) were seen but were not considered further.

For glycosylated SCR-16/20, the *c(s)* profile displayed significant monomer and minor dimer peaks, indicating that the largest SCR-16/20 fragment is mostly monomer. The *s*_*20,w*_ value for the monomer was 2.74 ± 0.02 S and that for the dimer was 4.75 ± 0.22 S (Fig. 4*C*). The masses were 44 ± 2.0 kDa for the monomer and 91.00 ± 1.0 kDa for the dimer, which are consistent with the sequence-derived monomer mass of 38 kDa. Post-deglycosylation, SCR-16/20 exhibited a slightly reduced monomer *s*_*20,w*_ value of 2.63 S and a reduced mass of 33.7 kDa. In terms of abundance, the monomer decreased from 86% to 81%, and the dimer also diminished from 8% to 2%. Overall, the changes are less pronounced for the five-SCR fragment.

For glycosylated SCR-16/18H, the *c(s)* profiles showed monomer and dimer peaks (Fig. 4*D*) with *s*_*20,w*_ values of 2.27 ± 0.33 S for the monomer and 3.85 ± 0.05 S for the dimer. The masses were 23.5 ± 4.0 kDa for the monomer and 54.25 ± 4.05 kDa for the dimer, being consistent with the sequence-derived monomer mass of 29 kDa. Integrations revealed 37% monomer and 63% dimer. In the case of SCR-16/18H De, the monomer peak was at 1.62 ± 0.12 S and the dimer at 2.83 ± 0.01 S. The masses also decreased to 17.6 ± 0.5 kDa and 41.4 ± 6.1 kDa, respectively, following deglycosylation. Overall, the changes are less pronounced for the three-SCR fragment, possibly because the digests of the three-domain SCR fragments impeded the removal of glycans by PNGase F.

### AUC of glycosylated and deglycosylated CFH

**Figure 5:**
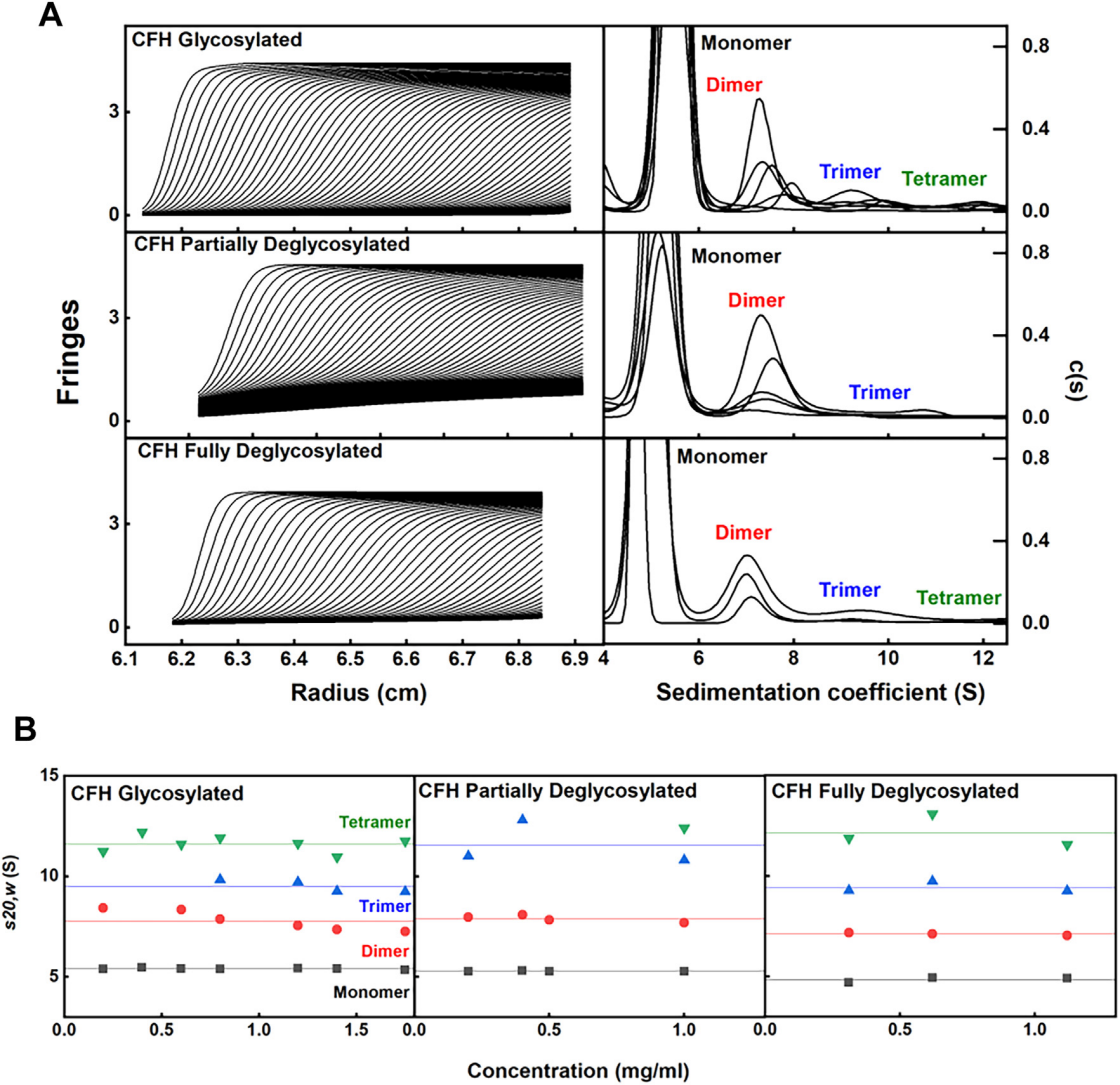
**Sedimentation velocity analyses for full-length CFH**. *A*, the experimental boundary fits for CFH, partially deglycosylated CFH, and fully deglycosylated CFH are shown in a concentration series in Hepes buffer from 0.2 mg/ml to 1.8 mg/ml. A total of 21 to 90 boundaries is shown from a total of 800 scans. The monomer, dimer, trimer, and tetramer peaks are labeled to correspond to the size distribution analyses *c(s)*. *B*, the concentration dependences of the *s*_*20,w*_ values for the monomer, dimer, trimer, and tetramer peaks are shown. The *s*^*0*^_*20,w*_ values of the glycosylated monomer and dimer are 5.57 ± 0.03 S and 8.12 ± 0.53 S, respectively, in Hepes buffer, those of the partially deglycosylated CFH monomer and dimer are 5.27 ± 0.02 S and 7.87 ± 0.17 S, and those for the fully deglycosylated CFH monomer and dimer are 5.02 ± 0.09 S and 7.21 ± 0.06 S. CFH, complement factor H.

The series of oligomers up to heptamers previously identified for native full-length CFH were re-examined by AUC (4, 7, 23). Here, native CFH, with concentrations ranging from 0.2 mg/ml to 1.8 mg/ml, displayed a sedimentation coefficient *s*_*20,w*_ for monomeric CFH at 5.57 ± 0.03 S, which agrees with the previous *s*_*20,w*_ values of 5.3 ± 0.1 S (4). The main peak corresponding to a mass of 171 ± 11 kDa for CFH (Fig. 5*A*) is consistent with the sequence-derived mass of 154 kDa. Integration revealed that the monomer and dimer peaks accounted for 96% and 4% respectively of glycosylated CFH. The deglycosylation of CFH resulted in the monomer peak shifting from 5.57 ± 0.03 S to 5.27 ± 0.02 S and to 5.02 ± 0.09 S in the partially and fully deglycosylated forms, respectively (Fig. 5*B* and Table 1). Likewise, the dimer peak shifted from 8.12 ± 0.53 S to 7.87 ± 0.17 S, then to 7.21 ± 0.06 S. These changes match those for the SCR fragments.

**Table 1:** Experimental AUC parameters for the SCR fragments and CFH

Protein sample	*s*_*20,w*_ (S) monomer	*s*_20,w_ (S) dimer	Proportion of monomer (%)	Proportion of dimer (%)	Monomer mass (kDa)	Dimer mass (kDa)
SCR-17H	1.90 ± 0.20	2.93 ± 0.04	33%	67%	14.9 ± 1.1	28.6 ± 2.5
SCR-17H deglycosylated	1.75 ± 0.26	NA^a^	96%	NA	8.8 ± 1.9	NA
SCR-18H	1.66 ± 0.22	3.82 ± 0.39	43%	57%	15.1 ± 2.1	35.6 ± 2.3
SCR-18H deglycosylated	1.56	NA	92%	NA	8.33	NA
SCR-17/18H	2.45 ± 0.01	3.81 ± 0.1	28%	72%	23.9 ± 1.1	44.5 ± 2.6
SCR-17/18H deglycosylated	2.27 ± 0.03	3.69 ± 0.01	84%	16%	20.1 ± 0.4	40.50 ± 1.56
SCR-17/18 *E. coli*	1.87 ± 0.13	NA	96%	NA	14.4 ± 1.0	NA
SCR-16/18H	2.27 ± 0.33	3.85 ± 0.05	21%	78%	23.5 ± 4.0	54.25 ± 4.05
SCR-16/18H deglycosylated	1.62 ± 0.12	2.83 ± 0.01	37%	63%	17.6 ± 0.5	41.4 + 6.1
SCR-16/20	2.74 ± 0.02	4.75 ± 0.22	86%	8%	44 ± 2.0	91.00 ± 1.0
SCR-16/20 deglycosylated	2.63	5.12	81%	2%	33.7	88.5
CFH glycosylated	5.57 ± 0.03	8.12 ± 0.53	96%	4%	171 ± 11	297 ± 21
CFH partially glycosylated	5.27 ± 0.02	7.87 ± 0.17	85%	15%	162 ± 9	293 ± 16
CFH fully deglycosylated	5.02 ± 0.09	7.21 ± 0.06	84%	16%	162 ± 5	278 ± 10

a NA, not available.

Overall, our AUC studies of the deglycosylated SCR fragments and CFH showed that the largest dimerization effects were seen for SCR-17/18H, SCR-17H, and SCR-18H. This work accounts for our previous study that identified SCR-17/18 as the principal dimerization location in the CFH C terminus (17). After deglycosylation, a dramatic decrease in dimerization was observed across the SCR fragments, in particular when *E. coli* SCR-17/18 was only seen as a monomer state. This reduction in dimerization was also evident in the full-length CFH sedimentation velocity experiments.

### SAXS of glycosylated and deglycosylated SCR fragments and CFH

**Figure 6:**
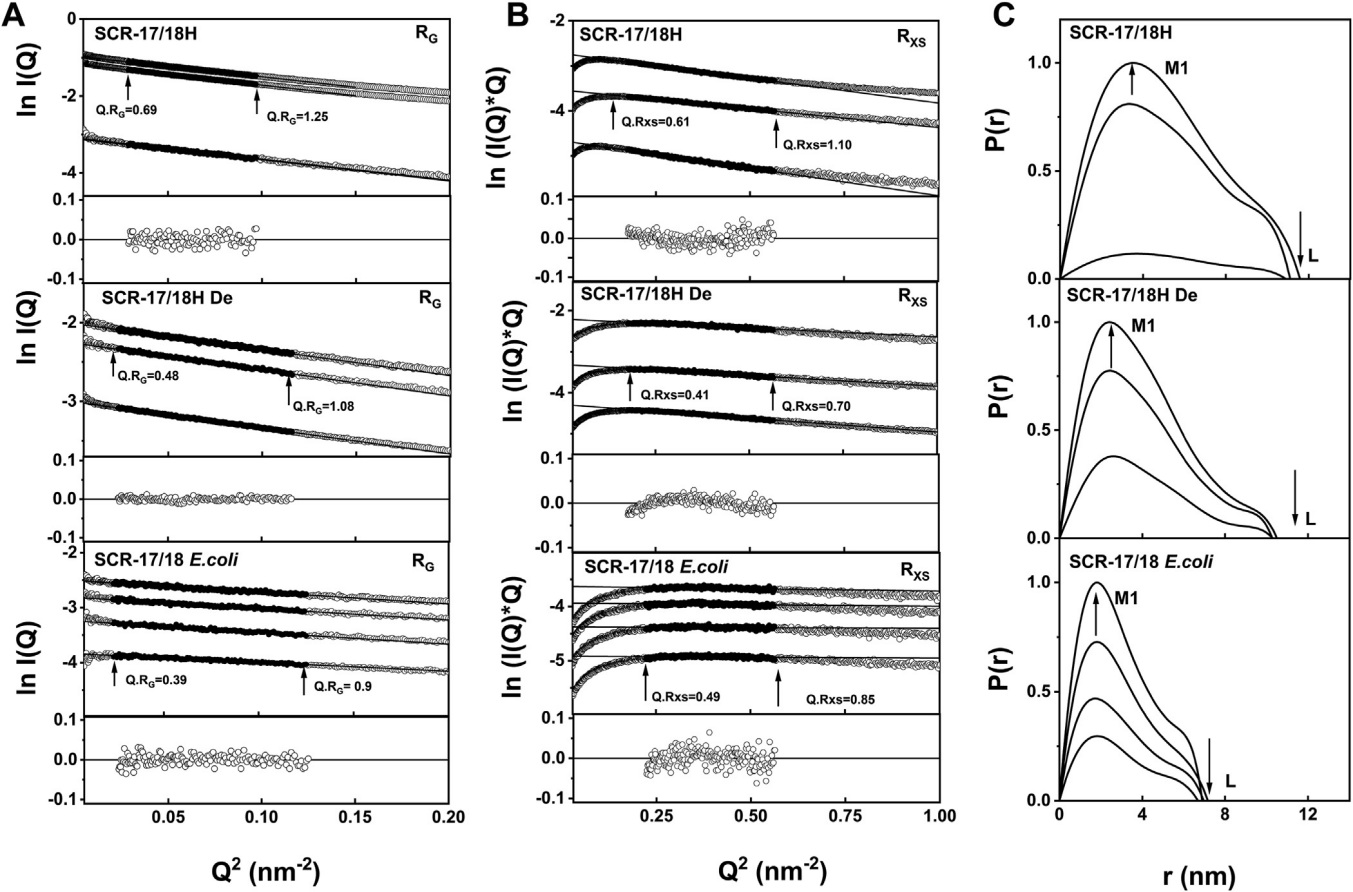
**X-ray analyses for the two-domain SCR-17/18 fragments.** Samples were measured in Hepes buffer. The filled circles correspond to the *I(Q)* values used to determine each *R*_*G*_ or *R*_*XS*_ value. Representative fit residuals are shown underneath each plot. *A*, Guinier *R*_*G*_ analyses for SCR-17/18H at 2.5 mg/ml, 2.0 mg/ml, and 0.6 mg/ml. The 0.6 mg/ml sample was run in SEC-SAXS mode while the two other concentrations were run in batch mode. The *Q* fit range was 0.17 to 0.31 nm^−1^. SCR-17/18H De was at 1.5 mg/ml, 1.0 mg/ml, and 0.6 mg/ml. The 0.6 mg/ml sample was run in SEC-SAXS mode while the other two concentrations were run in batch mode. The *Q* range was 0.15 to 0.34 nm^−1^. The SCR-17/18H *Escherichia coli* was at 4.0 mg/ml, 3.0 mg/ml, 2.0 mg/ml, and 1.0 mg/ml. The 1.0 mg/ml sample was run in SEC-SAXS mode while the other three concentrations were run in batch mode. The *Q* range was 0.15 to 0.34 nm^−1^. *B*, the corresponding Guinier *R*_*XS*_ analyses, using a *Q* range of 0.42 to 0.75 nm^−1^ for SCR-17/18. *C*, the corresponding *P(r)* analyses, where the arrows indicate the *M1* peak and *L* represents the maximum dimension of SCR-17/18. SAXS, small-angle X-ray scattering; SCR, short complement regulator.

**Figure 7:**
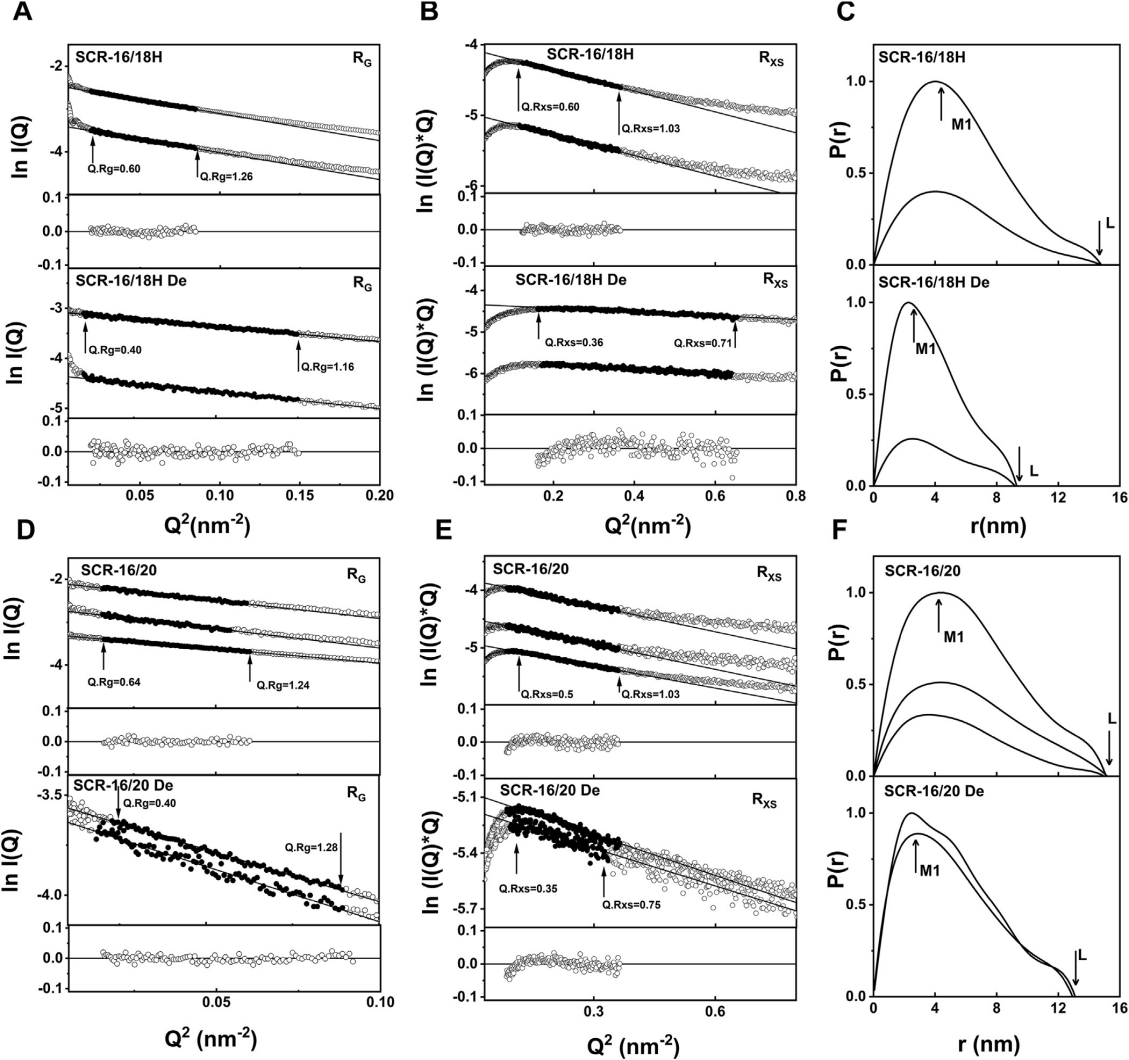
**X-ray analyses for the three- and five-domain SCR fragments of CFH.** Samples were measured in Hepes buffer. The filled circles correspond to the *I(Q)* values used to determine each *R*_*G*_ or *R*_*XS*_ value. Representative fit residuals are shown underneath each plot. *A*, Guinier *R*_*G*_ analyses for SCR-16/18H at 0.8 mg/ml and 0.4 mg/ml. The *Q* fit range was 0.14 to 0.29 nm^-1^. The SCR-16/18H De was at 0.5 mg/ml and 0.3 mg/ml. The *Q* fit range was 0.13 to 0.38 nm^−1^. *B*, the corresponding Guinier *R*_*XS*_ analyses, using a *Q* fit range of 0.35 to 0.60 nm^−1^ for SCR-16/18H and SCR-16/18H De. *C*, the corresponding *P(r)* analyses, where the arrows indicate the *M1* peaks and *L* represents the maximum dimension of SCR-16/18H and SCR-16/18H De. *D*, Guinier *R*_*G*_ analyses for SCR-16/20 at 1.1 mg/ml, 0.6 mg/ml, and 0.3 mg/ml. The *Q* fit range was 0.12 to 0.28 nm^−1^. The 0.3 mg/ml sample was run in SEC-SAXS mode, while the other concentrations were run in batch mode. The SCR-16/20 De was at 0.5 mg/ml and 0.4 mg/ml. The 0.3 mg/ml sample was run in SEC-SAXS mode while the other concentrations were run in batch mode. The *Q* fit range was 0.14 to 0.30 nm^−1^. *E*, the corresponding Guinier *R*_*XS*_ analyses, using a *Q* fit range of 0.30 to 0.60 nm^−1^ for SCR-16/20 and SCR-16/20 De. *F*, the corresponding *P(r)* analyses, where the arrows indicate the *M1* peaks and *L* represents the maximum dimension of SCR-16/20 and SCR-16/20 De. CFH, complement factor H; SAXS, small-angle X-ray scattering; SCR, short complement regulator.

**Figure 8:**
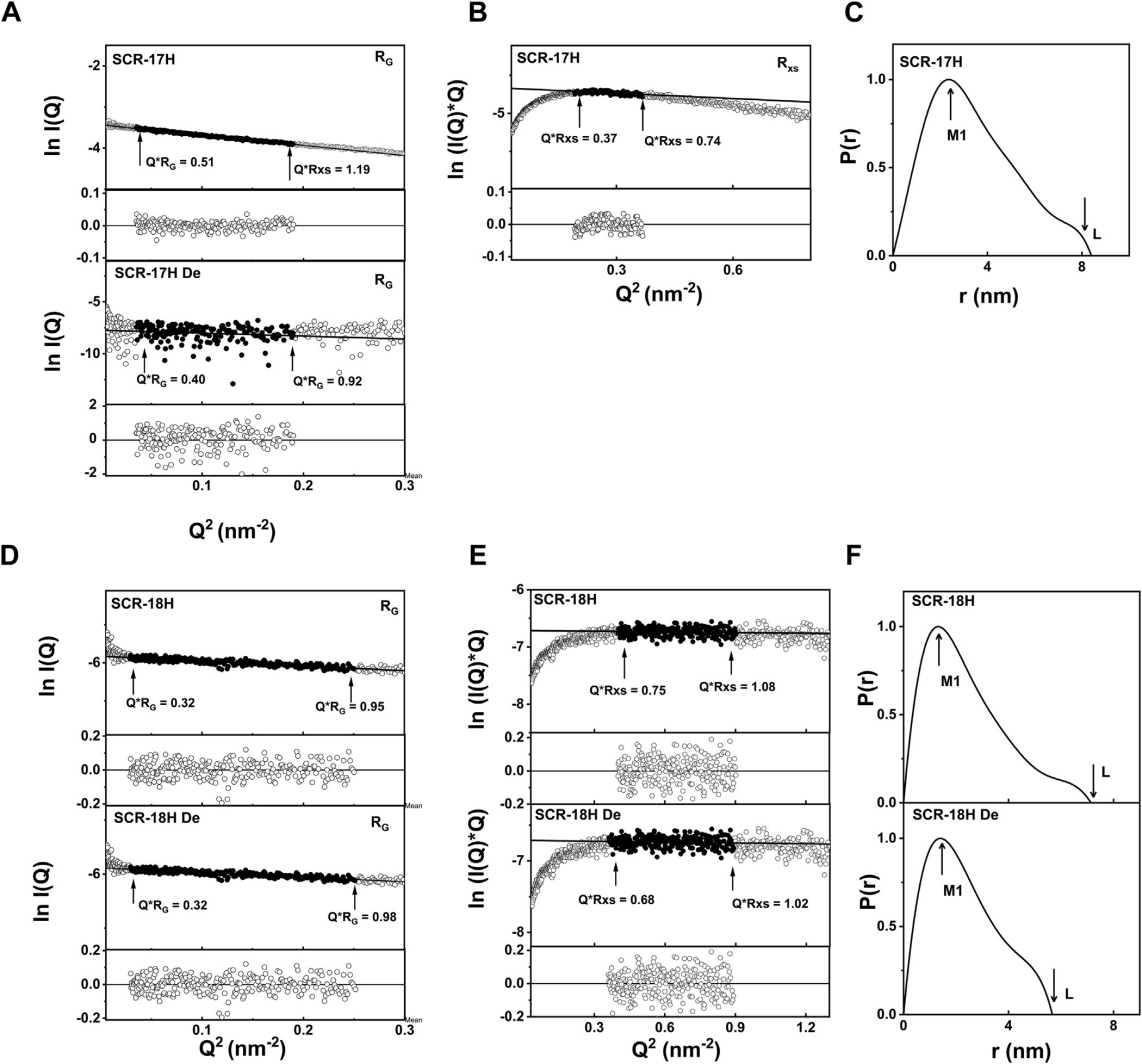
**X-ray analyses for SCR-17 and SCR-18 of CFH.** Samples were measured in Hepes buffer. The filled circles correspond to the *I(Q)* values used to determine each *R*_*G*_ or *R*_*XS*_ value. Representative fit residuals are shown underneath each plot. *A*, Guinier *R*_*G*_ analysis for SCR-17H at 0.4 mg/ml in SEC-SAXS mode. The *Q* fit range was 0.17 to 0.44 nm^−1^. SCR-17H De in Hepes buffer was at 0.1 mg/ml in SEC-SAXS mode. The *Q* fit range was 0.17 to 0.44 nm^−1^. *B*, the corresponding Guinier *R*_*XS*_ analysis, using a *Q* fit range of 0.46 to 0.60 nm^−1^ for SCR-17H. *C*, the corresponding *P(r)* analysis for SCR-17H, where the *arrows* indicate the *M1* peak and *L* represents the maximum dimension of SCR-17H. *D*, Guinier *R*_*G*_ analysis for SCR-18H at 0.3 mg/ml in SEC-SAXS mode. The *Q* fit range was 0.12 to 0.40 nm^-1^. The SCR-18H De in Hepes buffer was at 0.1 mg/ml in SEC-SAXS mode. The *Q* fit range was 0.13 to 0.44 nm^−1^. *E*, the corresponding Guinier *R*_*XS*_ analysis, using a *Q* fit range of 0.63 to 0.95 nm^−1^ for SCR-18H and SCR-18H De. *F*, the corresponding *P(r)* analysis, where the arrows indicate the *M1* peak and *L* represents the maximum dimension of SCR-18H De. CFH, complement factor H; SAXS, small-angle X-ray scattering; SCR, short complement regulator.

**Figure 9:**
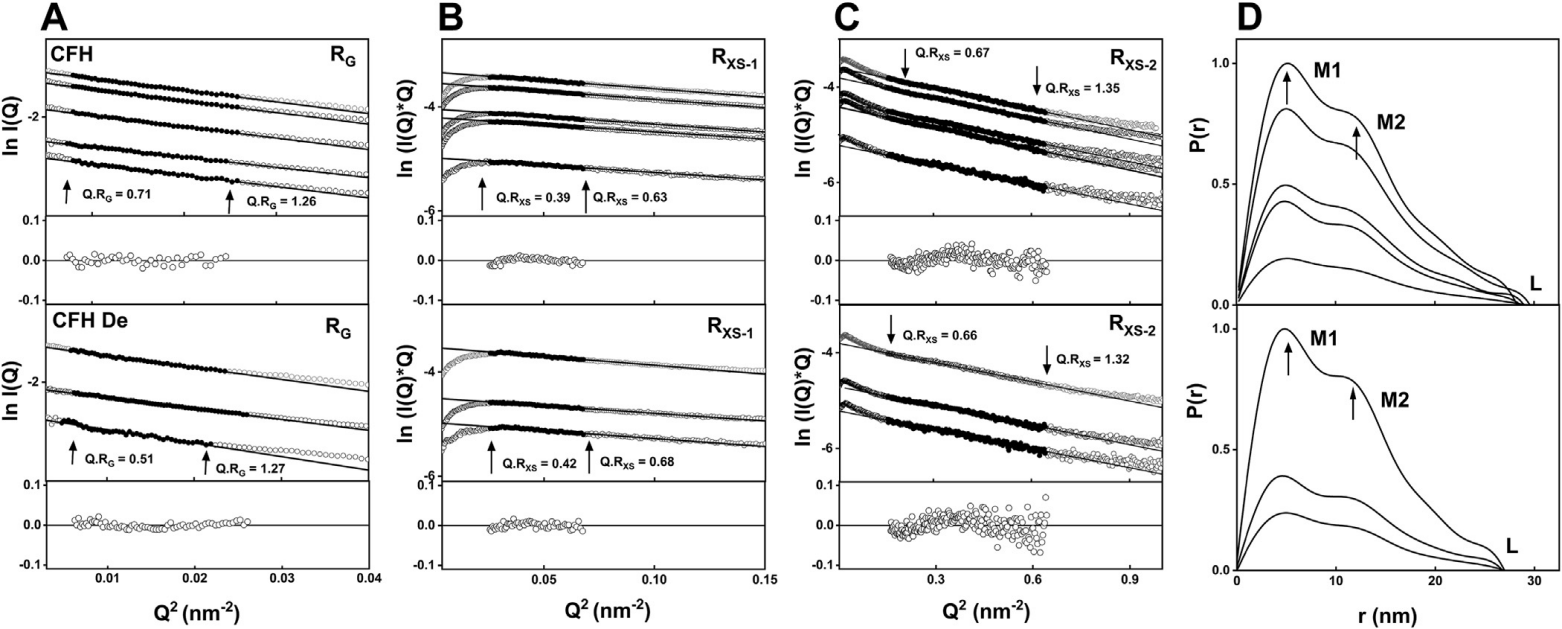
**X-ray analyses for full length CFH.** Samples were measured in Hepes buffer. The filled circles correspond to the *I(Q)* values used to determine each *R*_*G*_ or *R*_*XS*_ value. Representative fit residuals are shown underneath each plot. *A*, Guinier *R*_*G*_ analyses for native glycosylated CFH at 2.0 mg/ml, 1.5 mg/ml, 1.0 mg/ml and 0.4 mg/ml in batch mode, and 0.9 mg/ml in SEC-SAXS mode. The *Q* fit range was 0.07 to 0.12 nm^−1^. Fully deglycosylated CFH De was studied at 0.4 mg/ml, and 1.4 mg/ml in batch mode, and 0.75 mg/ml in SEC-SAXS mode. The *Q* fit range was 0.06 to 0.15 nm^−1^. *B*, the corresponding Guinier *R*_*XS-1*_ analyses, using a *Q* fit range of 0.16 to 0.26 nm^−1^ for CFH and CFH De. *C*, the corresponding Guinier *R*_*XS-2*_ analyses, using a *Q* fit range of 0.4 to 0.8 nm^−1^ for CFH and CFH De. *D*, the corresponding *P(r)* analyses, where the arrows indicate the *M1* and *M2* peaks and *L* represents the maximum dimension of CFH and CFH De. CFH, complement factor H; SAXS, small-angle X-ray scattering.

SAXS characterized the size and conformation of five SCR fragments and full-length CFH in Hepes buffer. High-quality SAXS data with excellent signal-to-noise ratios was acquired for most of the molecules under investigation (Figure 6, Figure 7, Figure 8, Figure 9). Guinier analyses were performed on the background-subtracted scattering curves to compute the radius of gyration (*R*_*G*_), indicative of the macromolecular overall elongation, and the cross-sectional radius of gyration (*R*_*XS*_) which clarifies the structural organization within the molecule. The Guinier analyses gave linear plots within acceptable fit limits for the observed *Q.R*_*G*_ and *Q.R*_*XS*_ ranges (24). The distance distribution function *P(r)* was also examined, which reports on the interatomic distances within the SCR fragments and CFH after Fourier transformation of the experimental scattering curve *I(Q)*. The maximum macromolecular *L* was deduced from the *r* value of the intersection of the *P(r)* curves with the abscissa at larger distances. The *r* value corresponding to the *P(r)* maximum gives the most prevalent interatomic distance within the samples and was indicated by *M1*.

For SCR-17/18H, its glycosylated and deglycosylated forms were examined in the concentration range of 0.6 mg/ml to 2.5 mg/ml. The Guinier analyses of the *I(Q)* curves yielded high-quality linear fits in two *R*_*G*_ and *R*_*XS*_ regions (Fig. 6*A*). For the glycosylated SCR-17/18H, the mean *R*_*G*_ value was 4.05 ± 0.02 nm, which decreased to 3.14 ± 0.01 nm following deglycosylation (Table 2). Concurrently, the mean *R*_*XS*_ value decreased from 1.45 ± 0.11 nm to 1.13 ± 0.06 nm (Table 2). SCR-17/18 from *E. coli* gave even lower *R*_*G*_ and *R*_*XS*_ values at 2.45 ± 0.13 nm and 0.74 ± 0.01 nm, respectively. These reductions are well explained by the loss of the dimeric structure following deglycosylation and the loss of the large glycan chains. The distance distribution function *P(r)* gave the maximum length *L* of glycosylated SCR-17/18H to be 11.2 ± 0.3 nm, which was reduced to 10.4 ± 0.1 nm after deglycosylation (Fig. 6*C*). The *M1* peak was 3.53 ± 0.02 nm, which decreased to 2.47 ± 0.01 nm following deglycosylation. Interestingly, the decrease in *M1* after deglycosylation is explained by the loss of the dimeric structure. For the *E. coli* sample, however, *M1* was reduced to 1.8 nm.

**Table 2:** Experimental SAXS parameters for the SCR fragments and CFH

Sample	Concentration (mg/ml)	R_G_ (nm)	R_XS_ (nm)	L (nm)	M1 (nm)	M2 (nm)
SCR-17/18H	2.5	4.07 ± 0.09	1.49	11.6	3.5	NA^a^
	2.0	4.03 ± 0.08	1.29	11.1	3.6	NA
	0.6^b^	4.05 ± 0.27	1.57	10.9	3.5	NA
SCR-17/18H without glycan	1.5	3.15 ± 0.11	1.08	10.5	2.5	NA
	1.0	3.13 ± 0.09	1.09	10.3	2.5	NA
	0.6^b^	3.15 ± 0.07	1.22	10.3	2.5	NA
SCR-17/18 *E. coli*	4.0	2.56 ± 0.26	0.75	6.9	1.8	NA
	3.0	2.48 ± 0.24	0.74	7.2	1.8	NA
	2.0	2.53 ± 0.27	0.72	7.0	1.8	NA
	1.0^b^	2.23 ± 0.43	0.75	6.7	1.8	NA
SCR-17H	0.4^b^	2.72 ± 0.12	1.1	8.4	2.4	NA
SCR-17H without glycan	0.1^b^	2.10 ± 4.7	1.0			
SCR-18H	0.2^b^	1.90 ± 0.47	1.2	7.1	1.3	NA
SCR-18H without glycan	0.2^b^	1.96 ± 0.39	1.1	5.7	1.4	NA
SCR-16/18H	0.8	4.35 ± 0.14	1.72	14.2	4.0	NA
	0.4	4.31 ± 0.28	1.71	14.2	4.1	NA
SCR-16/18H without glycan	0.5	2.99 ± 0.16	0.38	9.6	2.3	NA
	0.3	3.07 ± 0.47	0.86	9.3	2.4	NA
SCR-16/20H	1.1	5.00 ± 0.46	1.73	15.1	4.3	NA
	0.6	5.09 ± 0.92	1.72	15.2	4.4	NA
	0.3^b^	4.40 ± 0.60	1.61	15.7	3.2	NA
SCR-16/20H without glycan	0.5	4.05 ± 0.41	1.22	13.1	2.4	NA
	0.4^b^	3.90 ± 0.23	1.22	12.9	3.0	NA
CFH with glycan	2.0	8.85 ± 0.62	2.53	29.2	4.5	11.2
	1.5	8.20 ± 0.36	2.53	28.5	4.7	10.9
	1.0	8.11 ± 0.48	2.43	29.0	4.8	11.3
	0.4	8.00 ± 0.78	2.34	28.2	4.9	11.8
	0.9^b^	7.68 ± 0.42	2.36	28.6	4.8	11.5
CFH without glycan	1.4	8.25 ± 0.62	2.45	26.3	4.4	11.0
	0.4	8.76 ± 1.25	2.45	27.0	4.7	11.8
	0.75^b^	7.93 ± 0.27	2.40	26.9	4.2	10.0

a NA, not available.

b These values correspond to the SEC-SAXS runs.

For SCR-16/18H (three domains), the *R*_*G*_ and *R*_*XS*_ values were 4.33 ± 0.02 nm and 1.72 nm, respectively (Fig. 7, *A* and *B*). After deglycosylation, the *R*_*G*_ value decreased to 3.03 ± 0.04 nm and the *R*_*XS*_ value decreased to 0.622 ± 0.24 nm. The maximum dimension *L* decreased from 14.2 nm to 9.4 nm, which is in line with the loss of a dimeric structure (Fig. 7*C*). For SCR-16/20 (five domains), the *R*_*G*_ value decreased from 4.83 ± 0.3 nm to 3.9 ± 0.07 nm following deglycosylation. Concurrently, the *R*_*XS*_ value decreased from 1.68 ± 0.05 nm to 1.22 nm (Fig. 7, *D* and *E*). Accompanied by a reduction in the *L* values and changes in *M1* and *M2* values (Fig. 7*F*), these changed are explained again by a reduction in dimerization.

For the single domain SCR-17H and SCR-18H, the *R*_*G*_ and *R*_*XS*_ values were 2.72 ± 0.12 nm and 1.1 nm, respectively, and 1.90 ± 0.57 nm and 1.3 nm, respectively (Fig. 8). The SAXS data were noisier for reason of their small sizes and the low concentrations in use. However, following deglycosylation, the *R*_*G*_ and *R*_*XS*_ values were reduced to 2.10 ± 4.7 nm and 1.0 nm and a *R*_*G*_ value of 1.96 ± 0.39 nm and an *R*_*XS*_ value of 1.2 nm, respectively. While decreases were seen, the changes observed in SCR-18H and SCR-17H were more marginal when compared to the other larger SCR fragments.

For full-length CFH without and with full glycosylation (200 h) (Fig. 9), at concentrations ranging from 0.4 mg/ml to 2.0 mg/ml, averaged *R*_*G*_ values of 8.17 ± 0.38 nm for glycosylated CFH and 8.31 ± 0.34 nm for deglycosylated CFH were obtained (Table 2). Notably, these values were within error of each other, suggesting no significant difference in its folded back structure. The mean *R*_*XS-1*_ values were 2.44 ± 0.08 nm and 2.43 ± 0.08 nm (Table 2), calculated within acceptable Q.*R*_*XS*_ limits, and again within error. From the *P(r)* curves, after deglycosylation, the *L* value was slightly decreased from 28.7 ± 0.37 nm to 26.7 ± 0.30 nm, while the *M1* value was increased (but within error) from 4.41 ± 0.20 nm to 4.73 ± 0.12 nm, and the M2 value was increased also from 10.93 ± 0.72 nm to 11.34 ± 0.30 nm (but again within error). Unlike the smaller fragments of CFH, the full-length folded-back SCR structure of CFH was not much changed by deglycosylation.

### Atomistic modeling of the SCR fragments

Atomistic modeling aims to replicate the scattering curves of a macromolecule by testing randomized but physically realistic molecular models derived from Monte Carlo and molecular dynamics simulations until a best-fit structure emerges (25). Here, we modeled the SAXS data for five C-terminal SCR fragments using the recently published scattering model of full-length CFH to generate starting molecular structures (6). That study had produced 29,715 physically realistic, conformationally randomized structures for CFH. These resulted in the top 100 best-fit structures for the scattering curve of full-length CFH, as well as a single best-fit median CFH structure. Structures for the five SCR fragments were generated by modifying the median CFH structure. The C-terminal His-tags were modeled onto four of these fragments (Experimental procedures). Given that the SAXS data represents an average of the species present in the solution, models for both the monomer and dimer SCR fragments were utilized for fits with the experimental SAXS data. However, no accurate models for the CFH fragments dimers could be created for this comparison as these were not known nor could these be predicted accurately. By calculating the goodness-of-fit *R*-factor for each model in comparison with its experimental curves, the oligomeric state of each solution structure could be clarified.

**Figure 10:**
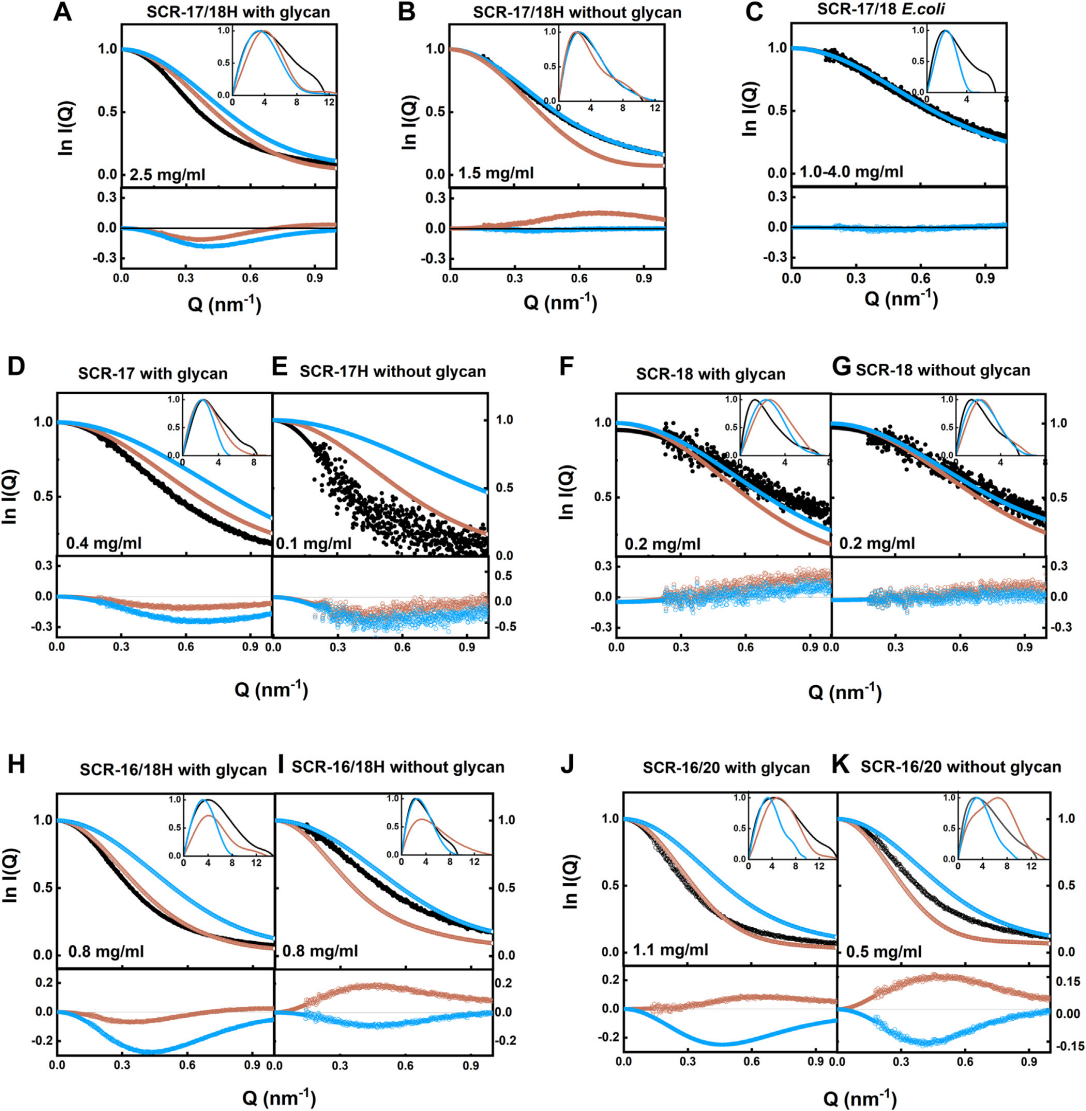
**Representative scattering curve fits to the experimental data for each of the best-fit SCR-17/18, SCR-17H and SCR-18H, and SCR-16/18H and SCR-16/20 best fit models**. Each experimental *I(Q)* and *P(r)* curve is denoted by *black circles* and *lines*, respectively, and each best-fit modeled curve is denoted by *solid blue lines* (monomer) or *red lines* (dimer). The curve fit residuals are shown below each fit. *A*–*C*, the modeled curve fits are shown for SCR-17/18H with two glycans, SCR-17/18H with no glycans, and SCR-17/18 from *Escherivhia coli*. *D–G*, the modeled curve fits are shown for SCR-17H with and without its glycan, and SCR-18H with and without its glycan, respectively. In *E*, the *P(r)* curve is not available due to poor data quality. *H–K*, the modeled curve fits are shown for SCR-16/18H with and without its glycan, and SCR-16/20 with and without its glycan, respectively. SCR, short complement regulator.

For SCR-17/18H, the modeling simulations were the most successful and confirmed that deglycosylation had removed the dimer form. For glycosylated SCR-17/18H, the fits for three different concentrations consistently showed that the dimer (red curves) provided a better fit to the data (black curves) than the monomer (green curves) (Fig. 10*A*). The dimer curve yielded a lower goodness-of-fit *R*-factor of 11%, compared to 22% for the monomer curve. This agreed with the AUC results (Fig. 4*A*), where both dimeric and monomeric SCR-17/18 were present in solution, with a relatively high amount of dimer formation. After deglycosylation, the monomer SCR-17/18H structure (green curve) showed a surprisingly good fit with the experimental SAXS data (black curves) at three concentrations, with an *R*-factor of 1.7 ± 0.4% (Fig. 10*B*). In contrast, the SCR-17/18H dimers gave an R-factor of 16.4 ± 0.5%. This outcome is consistent with the AUC results where more than 80% of the dimer was removed after deglycosylation. The SCR-17/18 from *E. coli* showed a similar result to that for the deglycosylated SCR-17/18H, with a low R-factor of 2.4 ± 0.6% against the experimental data (Fig. 10*C*).

For SCR-17H and SCR-18H, these were relatively small molecules with a molecular weight of only 14 kDa and poor signal-noise ratios. Reasonable visual fits (Fig. 10, *F* and *G*) were observed for SCR-18H with and without glycans. The fits for SCR-17H were less successful (Fig. 10, *D* and *E*).

For the two larger fragments SCR-16/18H and SCR-16/20, the curve fits showed qualitative agreements with the presence of dimer and monomer. AUC showed that SCR-16/18H was 60 to 80% dimeric, and the red curves (dimer) showed better agreement with the black curves than the green curves (monomer) (Fig. 10*H*). The dimer fit gave an R-factor of 7%, while the monomer gave a poorer R-factor of 37%. After deglycosylation, the monomer model (R-factor of 10%) fitted better than the dimer (21%) (Fig. 10*I*). This outcome confirms the AUC results, where dimerization was reduced after glycan removal. Similar to SCR-16/18H, SCR-16/20 shown that, after deglycosylation, the monomer model fitted better than the dimer model (Fig. 10, *J* and *K*). The R-factors for the dimer model were 10% and 20% before and after deglycosylation, respectively, while the R-factors for the monomer model were 35% and 17% before and after deglycosylation, respectively.

Overall, prior to deglycosylation, SCR-16/18H, SCR-16/20, and SCR-17/18H all demonstrated better fits with the dimer models. After deglycosylation, better fits were seen with the monomer model. The comparisons were less successful for SCR-17H and SCR-18H.

### SPR of glycosylated and deglycosylated SCR fragments and CFH

Having established the effect of glycosylation in CFH dimerization by AUC sedimentation velocity experiments and SAXS experiments and modeling, we now investigated the functional significance of CFH glycosylation in the CFH regulatory interaction with C3b. Two sets of SPR experiments determined the affinities of CFH self-association and the CFH–C3b interaction through the immobilization of one of the two proteins (the ligand) on a surface and measuring its interaction with its soluble partner (the analyte) (Table 3).

**Table 3:** Affinity of CFH and deglycosylated CFH binding to immobilized C3b and CFH

Protein sample	*K*_*D*_ (μM)	
	*C3b–CFH interaction*	CFH–CFH interaction
CFH glycosylated	0.90 ± 0.06	1.60 ± 0.63
CFH partially glycosylated	2.53 ± 0.42	NA^a^
CFH fully glycosylated	3.78 ± 0.45	11.50 ± 4.37

a NA, not available.

**Figure 11:**
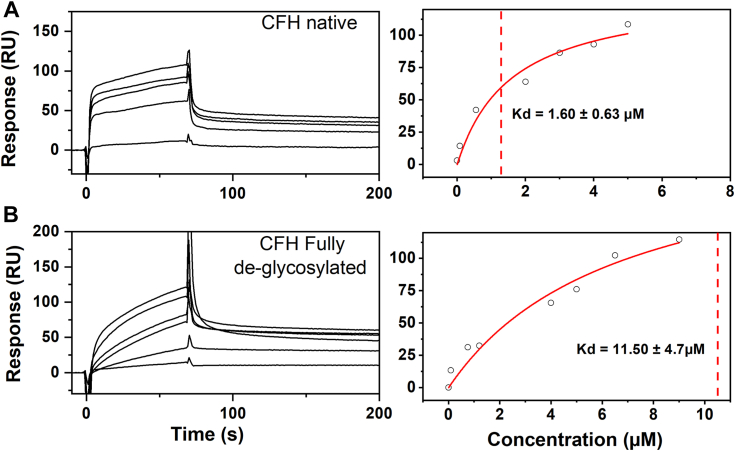
**Surface plasmon resonance study of the CFH-CFH self-association interaction using glycosylated and deglycosylated CFH**. The *left* panels show the sensorgrams for each CFH preparation interacting with a glycosylated CFH-immobilized SA sensor chip in Hepes buffer. The *right* panels show the corresponding *K*_*D*_ fits. The *K*_*D*_ values were calculated by fitting the reference-subtracted binding sensorgram to a 1:1 steady state affinity model. *A* and *B*, the two panels show the binding of glycosylated CFH and fully deglycosylated CFH in the concentration range of 0.01 μM to 1.8 μM in Hepes buffer interacting with a glycosylated CFH-immobilized chip. The fitted *K*_*D*_ values gave 1.60 μM and 11.50 μM, respectively. CFH, complement factor H.

The self-association of CFH, previously reported to occur under physiologically relevant conditions and featuring two dimerization sites of different affinities at two locations on CFH (4), was re-examined under a different SPR experimental setup. Previously, CFH was immobilized on CM3 chips, which might lead to nonspecific binding that would invalidate the runs. Here we used biotinylated native CFH which was immobilized on a streptavidin sensor chip to achieve a response unit (RU) of 781. Equilibrium experiments were performed in a Hepes buffer by flowing both native and fully deglycosylated CFH over the chip in a concentration range of 0.01 μM to 1.8 μM. For the self-association of native CFH with native CFH immobilized on the chip, a high-affinity K_D_ value of 1.60 μM was obtained (Fig. 11*A*). For the interaction between fully deglycosylated CFH and immobilized native CFH, the *K*_*D*_ value increased to 11.50 μM (Table 3), indicating a ten-fold decrease in affinity (Fig. 11*B*). This outcome provided further evidence (in addition to AUC and SAXS above) for the importance of glycosylation in mediating the self-association properties of CFH.

**Figure 12:**
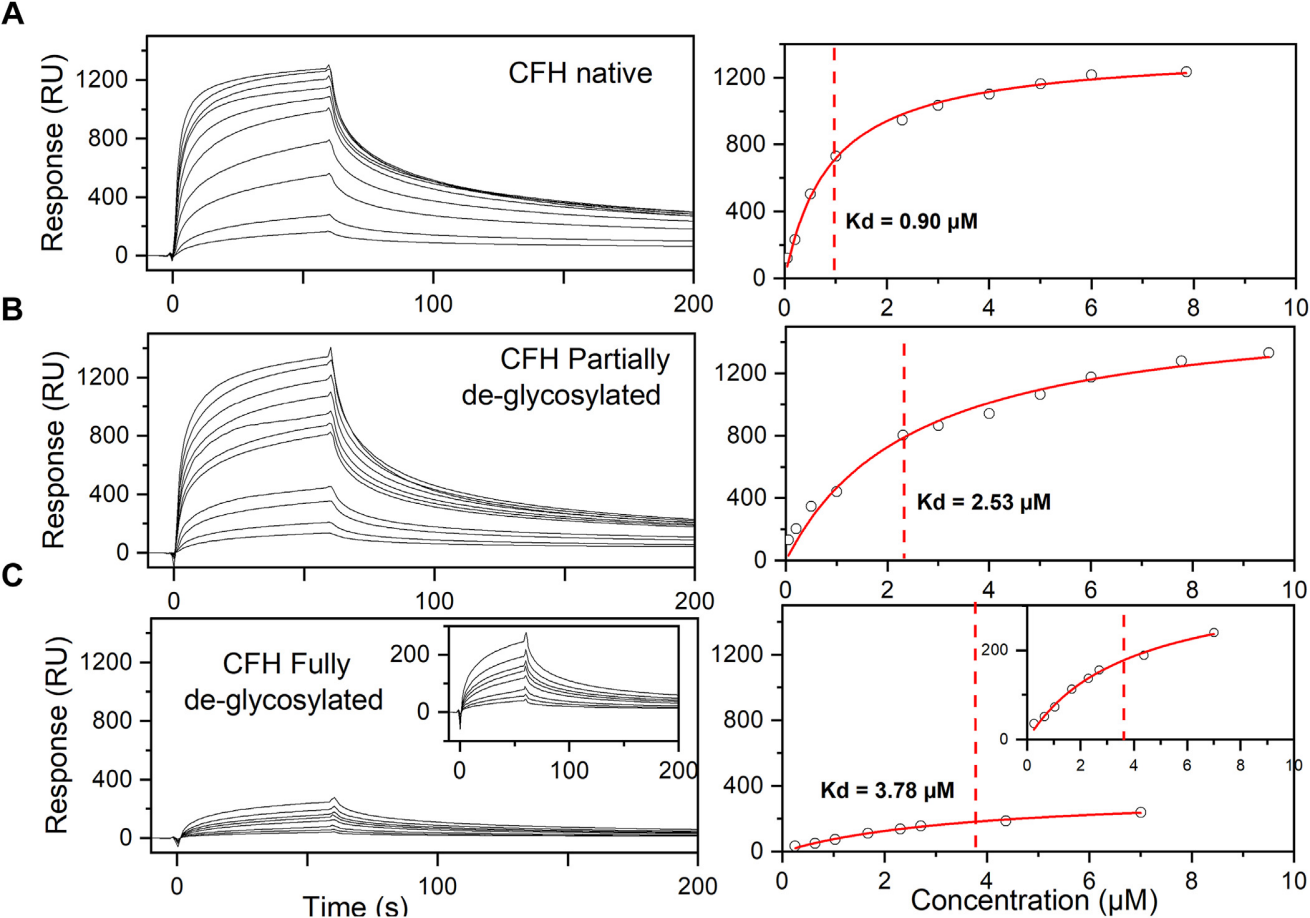
**Surface plasmon resonance study of the C3b–CFH interaction with CFH and deglycosylated CFH**. The *left* panels show the sensorgrams for each of the three CFH preparations that measured its affinity with a C3b-immobilized CM5 sensor chip in Hepes buffer. The *right* panels show the corresponding *K*_*D*_ binding fits. The *K*_*D*_ values were calculated by fitting the reference-subtracted binding sensorgrams to a 1:1 steady state affinity model. *A–C,* the three panels show in that order the interaction of WT native glycosylated CFH, partially deglycosylated CFH, and fully deglycosylated CFH at concentrations of 0.05 μM to 7.75 μM in Hepes buffer with C3b immobilized on a CM5 chip. The fitted *K*_*D*_ values were 0.9 ± 0.06 μM, 2.53 ± 0.42 μM, and 3.78 ± 0.45 μM in that order. The inset in the *bottom* panel represents an expanded view of the sensorgram. CFH, complement factor H.

For the CFH–C3b interaction, purified C3b (Experimental Procedures) was immobilized onto CM5 chips until an immobilization level of 12,349 RU was reached. Equilibrium experiments were then executed with native, partially deglycosylated, and fully deglycosylated CFH (Fig. 12). Each CFH preparation was flowed over the chip across a range of concentrations (0.05 μM to 7.75 μM) in a Hepes buffer. Notably, at all concentrations, an interaction between CFH and the immobilized C3b was observed when CFH was in its native state. A *K*_*D*_ value of 0.9 μM was calculated from a 1:1 steady-state affinity analysis (Table 3), which agreed with previous work that indicated a *K*_*D*_ value of 1.06 μM (26). (Fig. 12*A*). However, as the glycan moieties were removed from CFH during 100 h and 200 h of digests, the *K*_*D*_ values increased to 2.53 μM and 3.78 μM (Table 3 and Fig. 12, *B* and *C*). This showed that the CFH affinity for C3b decreased by two-to three-fold following deglycosylation. Given that the C terminus of CFH binds to C3b, the diminished affinity was attributed to the role of CFH glycosylation in increasing the local concentration of native CFH at the C3b-immobilized surface to enhance the CFH interaction with C3b.

## Discussion

For the first time, we report the novel finding that the high glycosylation of CFH affects its ability both to form weak dimers and to regulate the activity of complement C3b. Our observations are consistent with dimer formation between SCR-17/18 stabilized by either intermolecular glycan–glycan or glycan–protein interactions. The functional significance of such glycan–glycan or glycan–protein interactions have previously been reported. A prominent example is the two conserved glycans in the Fc region of human IgG1 antibodies. Their removal causes the antibody Fc region to exhibit a broader and more flexible range of conformations than the WT glycosylated structure and led to disorder in the Fc structure that in turn affected binding to Fc receptors (19). The observation of CFH oligomers has a long history. Full-length CFH forms weak dimers, with an estimated 4 to 15% dimer and higher forms present at typical CFH serum concentrations of 0.8 to 3.6 μM (0.116–0.562 mg/ml) (27). The dissociation constants (*K*_*D*_ values) for dimer formation range between 8 to 28 μM, suggesting that CFH dimers coexist with CFH monomers under physiological serum conditions. Previous studies have identified two separate CFH self-association sites in the SCR-6/8 and SCR-16/20 regions (23, 28). The dimer sites lead to the formation of daisy-chains of oligomeric CFH. Subsequently, the SCR-19/20 domain pair was ruled out as the dimer site in SCR-16/20, which was then more precisely located to SCR-17/18 (17). There, a combination of AUC and SAXS experiments, along with molecular simulations based on our recent full-length CFH model (6), showed that SCR-17H forms more dimers than SCR-18H. In this study, by preparing the SCR-17/18 domain pair in *E. coli* which was then devoid of glycans and suitable for crystallography, we applied the same strategy as in (17) to discover unexpectedly that glycan-free SCR-17/18 did not dimerize. This finding was explored further here using AUC and SAXS studies to determine the solution structure of five SCR fragments that included SCR-17/18, both with and without glycosylation. The results consistently showed that the removal of the SCR-17/18 glycans reduced the dimerization of these fragments. The study of SCR-16/20 showed that the His-tag did not affect dimerization.

**Figure 13:**
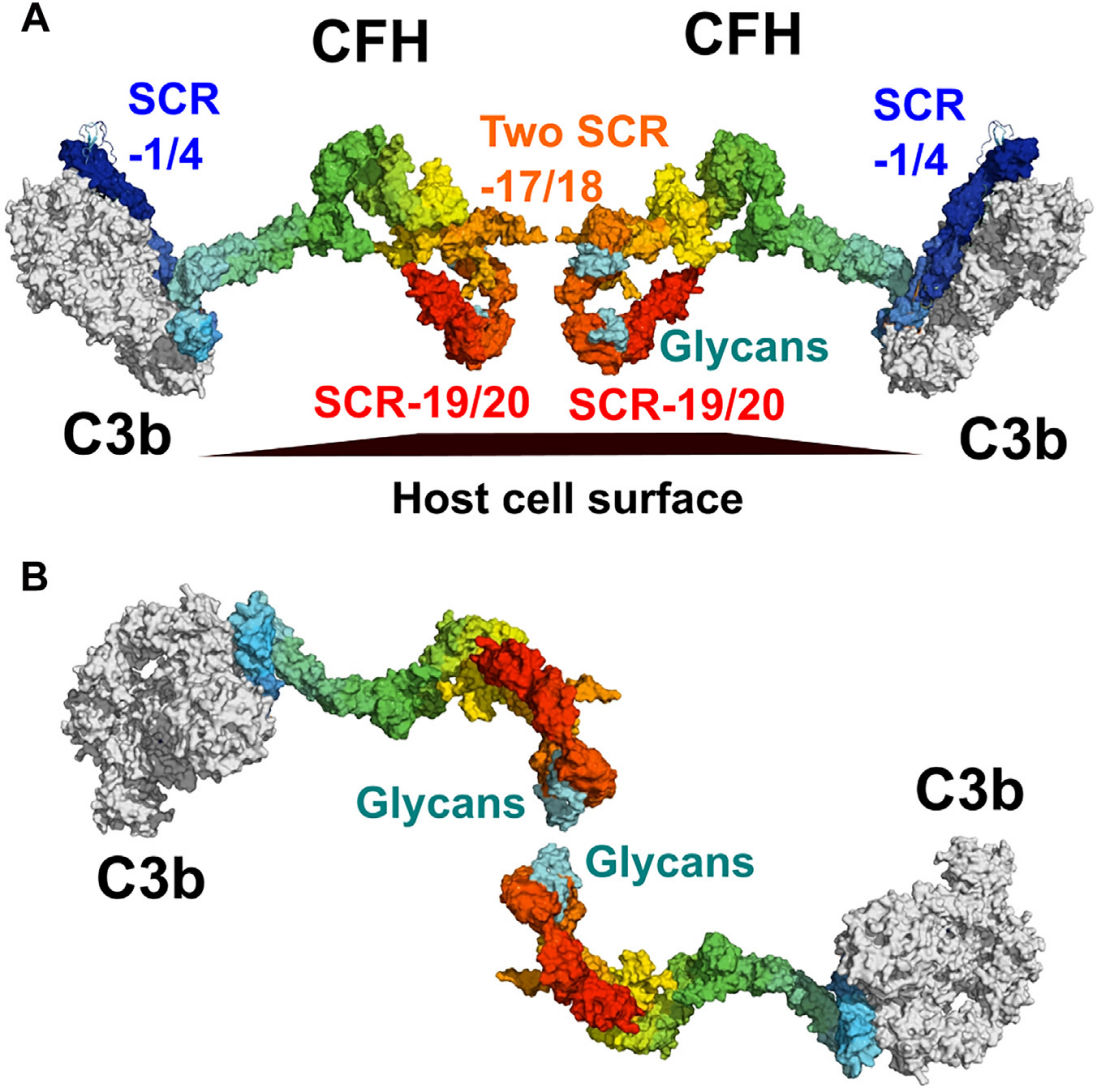
**Schematic diagrams of a proposed assembly of a dimeric CFH complex with complement C3b at host cell surfaces**. *A,* C3b is shown bound to CFH at SCR-1/4. The dimer interface is shown as two molecules of SCR-17/18 (*orange*) in proximity to each other. The two glycan chains on SCR-17 and SCR-18 are shown in *cyan*, although only two chains of the four are fully visible in the orientation as shown. SCR-19/20 (*red*) is shown in proximity to the host cell surface with which it interacts through anionic polysaccharides or with covalently bound C3d fragments of C3b. To make this figure, the crystal structure of the C3b complex with SCR-1/4 of CFH (PDB ID: 2WII) was superimposed onto one of the best-fit scattering models of full-length CFH (6, 12). *B*, the view in (*A*) is rotated by 90° and the two molecules are positioned nearer to each other at the center to show how the four glycans might interact with each other to promote CFH dimerization. An alternative interaction may involve protein-glycan contacts that promote dimerization. CFH, complement factor H; SCR, short complement regulator.

The glycosylation of CFH affects the native full-length structure. Full-length CFH showed dimerization extents of 4 to 15% (27), while the smallest CFH fragments showed up to 80% dimerization (Fig. 4*B*). The reduced dimer formation for larger CFH molecules is attributed to steric effects caused by their larger sizes, which are presumed to inhibit CFH dimer formation. A remarkable feature of the native CFH glycosylation is the length of time of up to 200 h required for PNGase F to cleave its eight glycan chains from CFH. In the human IgG1 and IgG4 subclasses, the same deglycosylation reaction with PNGase F only required 10 h for the full deglycosylation of the two glycans in its Fc region (19, 29). A similar period of 12 h was required to remove the two glycans from *Pichia*-expressed SCR-17/18 in the current study. The ability of the SCR-17/18 glycans to mediate dimer formation implies that these are surface-exposed and may be more accessible to PNGase F cleavage (Fig. 13). The other six glycans were located at SCR-9 to SCR-15, and these correspond to the core of the folded-back CFH protein structure, which presumably hinder the access of the enzyme PNGase F to SCR-9 to SCR-15. Interestingly, the overall solution structure from the SAXS experiment showed little change following deglycosylation. It appears likely that, during synthesis, the folded-back SCR structure of full-length CFH is formed first, then this structure is stabilized by the addition of around six glycan chains at strategic locations in the core of the CFH structure.

As the major complement regulator, CFH protects host cells from C3b-mediated destruction through its C-terminal binding to C3b, C3d, and anionic host cell surfaces. The C-terminal dimerization of CFH (Fig. 13) would promote a higher CFH concentration on host surfaces during an inflammatory response to protect this better. The binding of SCR-1/4 to C3b would be augmented, as depicted in Figure 13 (12). The current study shows the importance of glycosylation for this. This functional importance was demonstrated by our SPR investigation, when, after removing glycans, the CFH affinity for C3b was significantly reduced (Fig. 12*B*). Overall, these findings could represent a self-regulation mechanism for CFH to maintain an optimal concentration of dimeric CFH in plasma and for CFH dimers to increase their abundance at host cell surfaces for reason of proximity arrangements of both the SCR-17/18 glycans to a neighboring CFH molecule and to polyanions or surface-bound C3d molecules that bind to CFH at SCR-19/20. Such a mechanism would promote the further binding of CFH molecules at host cell surfaces in order to increase the protection against active C3b molecules in the vicinity. This improved understanding of CFH glycosylation may open the way for the development of more therapeutically effective CFH recombinant molecules. The molecular modeling of CFH dimers (Fig. 13) also provided a new functional explanation for the formation of SCR-17/18 dimers and insight into why aHUS disease-associated genetic variants occur along the length of SCR-16/20 and not just in SCR-20 (Fig. 10) (13, 30). Such variants may disrupt the orientation of the protein structures that are required for effective SCR-17/18 dimer formation.

## Experimental procedures

### Purification of six SCR fragments, CFH and C3b

The expression and purification of one-to five-SCR fragments of CFH in the five constructs SCR-16/20, SCR-16/18H, SCR-17/18H, SCR-17H, and SCR-18H in a *P. pastoris* expression system were as described elsewhere (17). The suffix H denotes a hexa-Histidine tag. The sequences at the N- and C- termini of the five SCR fragments varied with the specific fragment. For all five, their N-termini incorporated the EAEAF sequence, which correlates with the α-factor secretion signal and the *EcoRI* restriction enzyme site. The four SCR-18H, SCR-17H, SCR-17/18H, and SCR-16/18H C-termini consisted of the first four amino acids from the succeeding linker region, followed by the *myc* tag and His tag sequences ALEQKLISEEDLNSAVDHHHHHH. The SCR-16/18H and SCR-16/20 fragments contain the first three residues of the next linker. The *Pichia* expression plasmid pPICZaA served as a cloning vector for the SCR domains and was subsequently transformed into WT X33 cells to follow Invitrogen's guidelines (17). Selected transformants were selected using zeocin, given the encoding of a zeocin-resistant gene by pPICZaA (20). The cells were cultured in a medium supplemented with 2% glycerol for 4 days. The induction of recombinant protein expression utilized 0.5% methanol and was repeated every 24 h for 4 days. Postcentrifugation of the cells, the supernatant with the secreted SCR domains was concentrated using a 5 kDa molecular weight cutoff membrane.

The four His-tagged fragments (SCR-17H, SCR-18H, SCR-17/18H, and SCR-16/18H) were purified using a 5 ml HiTrap Nickel column (Cytiva). The supernatant was dialyzed against a wash buffer (50 mM NaH_2_PO_4_, 300 mM NaCl, 10 mM imidazole, pH 8.0) and loaded onto an AKTA Purifier system (Cytiva). To remove any proteins bound nonspecifically to the HiTrap column, the column was washed with five volumes of wash buffer. The proteins were then eluted using 50 mM NaH_2_PO_4_, 300 mM NaCl, 250 mM imidazole, pH 8.0. For SCR-16/20 without a His-tag, cation exchange chromatography was employed, given the theoretical isoelectric point of 8.04 for SCR-16/20. Following dialysis of the supernatant against 50 mM Tris–HCl, 25 mM NaCl, 1 mM EDTA, pH 7.4, this was loaded onto a SP FF column (Cytiva) previously equilibrated with the same buffer. The column was then washed with five column volumes of buffer and the protein was eluted *via* a salt gradient up to 1 M NaCl.

To express SCR-17/18 in *E. coli*, the SCR-17/18 plasmid was designed and procured (Genscript Biotech), utilizing the pET-21a expression vector. The plasmid was sequenced between the T7 forward and M13 reverse primers and validated against the SCR-17/18 sequence. The recombinant plasmid was transformed into BL21 DE3 competent *E. coli* cells using heat shock. BL21 DE3 cells, 100 μl, were thawed on ice and incubated with recombinant DNA for 30 min. Subsequently, a heat shock treatment at 42 °C was applied for 30 s. The cell–DNA mixture was swiftly transferred to ice for 3 min, followed by the addition of 250 μl of super optimal medium (2.0% tryptone, 0.5% yeast extract, 10 mM NaCl, 2.5 mM KCl, 10 mM MgCl_2_, 10 mM MgSO_4_, 20 mM glucose) for cell growth at 37 °C for an hour. To isolate the transformed cells, they were cultured on LB-agar plates containing ampicillin, followed by an overnight incubation at 37 °C. The cells that survived this selection process were then transferred to small-scale incubation flasks with 20 ml LB, maintained at 37 °C, and agitated at 220 rpm to initiate a starter culture. Following an overnight growth, 5 ml of the SCR-17/18 starter culture was utilized for a miniprep to sequence the plasmid, while another 5 ml was used for an induction test to ensure that the right protein was being produced post-IPTG induction. The remaining 10 ml of starter culture was transferred to a larger 500 ml LB medium for further growth. Cell growth was monitored from the optical density at 600 nm using a Genesys 10 Bio UV-visible spectrophotometer (Thermo Fisher Scientific). Upon reaching an optical density of 0.6, IPTG was added to the medium to a final concentration of 1 mM, followed by a further incubation for 4 h, after which the cells were harvested by centrifugation at 9000 g for 30 min at 4 °C. The cell pellets were re-suspended in 40 ml of lysis buffer containing 20 mM Tris, 1 mM EDTA, pH 8.0, alongside an EDTA-free protease inhibitor cocktail tablet (Roche) and 0.5 mg/ml of Pefabloc SC (Sigma-Aldrich). A subsequent addition of 10 ml of 0.4% (w/v) sodium deoxycholate buffer was made, followed by an hour-long incubation. Cell lysis was achieved by sonication and the resultant homogenate was centrifuged at 10,000g for 40 min at 4 °C. The resulting pellet, containing the inclusion bodies, was washed twice with Dulbecco’s phosphate-buffered saline enriched with 0.05 Tween20.

To purify SCR-17/18 without a His-tag, cation exchange chromatography was employed, based on a calculated isoelectric point of 7.56 for SCR-17/18. Following dialysis of the supernatant against 50 mM Tris–HCl, 25 mM NaCl, 1 mM EDTA, pH 6.5, this was loaded onto an SP FF column (Cytiva) previously equilibrated with the same buffer. The column was then washed with five column volumes of buffer and the protein was eluted *via* a salt gradient up to 1 M NaCl. Eluted protein was then concentrated with 3 kDa MWCO centrifugal filter (Amicon, Sigma) and subjected to a final round of size-exclusion chromatography using a Superose 16/200 prep grade column with a 10 mM Hepes, 137 mM NaCl, pH 7.4 buffer.

To purify full-length CFH, a stock of frozen human plasma genotyped for the WT Tyr402 polymorphism was thawed from −80 °C to 4 °C overnight and then dialyzed overnight in a 2 L running buffer (25 mM Tris–HCl, 140 mM NaCl, 0.5 mM EDTA-Na_2_, pH 7.4). Following dialysis, the plasma was centrifuged for 15 min at 11,000 rpm to remove precipitates. The resulting supernatant was passed through a membrane with a pore diameter of 0.22 μm. Next, three columns were used in succession at 4 °C, specifically a nonimmune IgG-immobilized Sepharose column, a L-lysine Sepharose affinity column, and a monoclonal MRC-OX23 Sepharose affinity column (6, 31). The initial column was a nonimmune IgG column to eliminate plasma proteins that bind to immunoglobulins. The next column was a Lysine-Sepharose column to remove plasminogen and plasmin. The monoclonal MRC-OX23 column binds CFH, which was eluted with 3 M MgCl_2_ at pH 6.9. The optical density at 280 nm was used to verify that CFH had been eluted. After elution, the CFH was dialyzed against Hepes buffer (10 mM Hepes, 137 mM NaCl, pH 7.4) with 0.5 mM EDTA added to remove MgCl_2_. Post-dialysis, CFH was further purified using a HiTrap Protein G column to remove any remaining IgG contaminants. CFH was then concentrated to 3 to 4 mg/ml with a 50 kDa MWCO membrane.

To purify C3b, complement C3 was first isolated from fresh human plasma *via* a slightly modified protocol based on (32). Initially, the plasma, supplemented with EDTA and AEBSF protease inhibitor (Thermo Fisher Scientific), was gently mixed with polyethylene glycol (PEG 3350) to precipitate larger plasma proteins. The supernatant was treated with an increased concentration of PEG 3350 and centrifuged. The protein pellet was resuspended in a buffer and subjected to ion-exchange chromatography using a HiPrep QFF 16/10 column. Nonspecific proteins were removed through a wash step, and C3 was selectively eluted *via* a linear salt gradient. The eluted C3 was further processed *via* a Mono Q 5/50 Gl column, following its dilution to reduce salt concentrations. The final purification stage involved concentrating the protein and subjecting it to size-exclusion chromatography *via* a Superose 6 prep grade XK 16/60 column. C3b was generated by incubating C3 with trypsin for a strictly maintained duration of 120 s at 37 ºC. The reaction was halted by the addition of 40 μg/ml trypsin inhibitor (from *Glycine max*), following which, iodoacetamide was added to block the free thiol group in C3b. This mixture was kept in the dark at 20 ºC for 30 min (30). Subsequently, C3b was diluted in Tris buffer (25 mM Tris, 140 mM NaCl, 0.5 mM EDTA, pH 8.0), concentrated, and subjected to a final round of size-exclusion chromatography using a Superose 6 prep grade XK 16/60 column.

### Deglycosylation and composition of the SCR fragments and CFH

Prior to measurements (below), the seven samples (SCR-16/20, SCR-16/18H, SCR-17/18H, SCR-17H, and SCR-18H from *Pichia*, SCR-17/18 from *E. coli*, and CFH from plasma) were each processed using size-exclusion chromatography with a prepacked Superdex 200 10/300 Gl column (Cytiva) (Fig. 2*A*) to eliminate nonspecific aggregates. All five *Pichia*-expressed SCR fragments and CFH were deglycosylated using PNGase F (New England Biolabs) (5, 20, 21). The five glycosylated SCR fragments were mixed with 1 μl of 10,000-unit PNGase F and incubated for 24 h at 37 °C. Native CFH was mixed with 1 to 10 μl of 10,000-unit PNGase F and incubated from 100h to 200h at 37 °C. Gel filtration was used to remove the unwanted PNGase F.

For mass spectrometry (Fig. 3), the SCR fragments and CFH were concentrated and buffer-exchanged into PBS using Amicon Ultra-0.5 3 kDa to 50 kDa cut-off centrifugal filters depending on the protein molecular weight to achieve a concentration of 0.2 mg/ml. All samples at 0.2 mg/ml, native and deglycosylated, were buffer-exchanged 2 hours prior into 100 mM ammonium acetate using Zeba desalting columns. Mass spectrometry was conducted on an Agilent 6510 quadrupole time-of-flight liquid chromatography system. The 8 μl samples were injected into a PLRP-S, 1000 A column measuring 150 mm × 2.1 mm. The column was kept at 60 °C with a flow rate of 0.3 ml/min. To achieve separation, two mobile phases A (water with 0.1% formic acid) and B (acetonitrile, with 0.1% formic acid) were employed, utilizing gradient elution. The column output was continually electrosprayed into the capillary electrospray ionization source of the Agilent 6510 spectrometer. The ionization mass spectra were collected in the positive electrospray ionization mode, using the mass/charge *m/z* range of 1000 to 3200 in profile mode. The spectra were analyzed using Mass Hunter software version B.07.00, equipped with an inherent protein deconvolution function to transform zero-charge mass spectra into intensity/mass plots.

### AUC data collection and analyses for the SCR fragments and CFH

AUC sedimentation velocity data for each glycosylated and deglycosylated SCR fragment and CFH in Hepes buffer were acquired at 20 °C, utilizing an AnTi50 rotor in an Optima AUC instrument (Beckman Coulter). Rotor speeds of 50,000 rpm for the SCR fragments and 40,000 rpm for CFH were used. Concentrations ranged between 0.2 to 3 mg/ml. Interference and absorbance optics at 280 nm were employed for detection purposes in 10 to 15 h runs; note that the absorbance data showed saturation at higher concentrations. The sedimentation boundaries from 300 to 600 scans were analyzed using the *c(s)* size distribution model in SEDFIT software to give sedimentation coefficients, corrected to standard *s*_*20,w*_ values, (33, 34). Typically, 40 to 100 boundaries were analyzed. For the fits, the overall RMSD was deemed acceptable if it was less than 0.02, following adjustments of parameters including the meniscus and cell bottom positions, the baseline, and the frictional ratio *f/f*_*0*_. This resulted in size-distribution *c(s)* analyses that depicted the sedimentation coefficient(s) *s*_*20,w*_ for the species present. To measure the Hepes buffer density and viscosity at 20 °C, a DMA 5000 density meter and a Lovis microviscometer were employed (Anton Paar). The buffer density was 1.00478 g/ml and the buffer viscosity was 1.020 mPa s.

The partial specific volume $\bar{v}$ values for each SCR fragment and CFH, both glycosylated and deglycosylated, was computed from their sequence-determined compositions (35). The absorption coefficients (280 nm, 1%, 1 cm) for concentration determinations were likewise calculated from the compositions (35). The calculated $\bar{v}$ values were as follows: for CFH and deglycosylated CFH, they were 0.7154 ml/g and 0.7242 ml/g with absorption coefficients of 15.9 and 18.0, respectively; for SCR-17H and its deglycosylated form, they were 0.7035 ml/g and 0.7181 ml/g, with absorption coefficients of 9.2 and 12.0, respectively; for SCR-18H and its deglycosylated form, they were 0.7019 ml/g and 0.7139 ml/g, with absorption coefficients of 9.2 and 11.4, respectively; for SCR-17/18H, its deglycosylated form, and the *E. coli* variant, they were 0.7030 ml/g, 0.7184 ml/g, and 0.7182 ml/g, respectively, with absorption coefficients of 11.1, 14.3, and 16.3, respectively; for SCR-16/18H and its deglycosylated form, they were 0.7079 ml/g and 0.7200 ml/g, with absorption coefficients of 12.0 and 14.8, respectively; for SCR 16/20 and its deglycosylated form, they were 0.7144 ml/g and 0.7231 ml/g, with absorption coefficients of 15.4 and 16.3, respectively.

### SAXS data collection and analyses for SCR fragments and CFH

SAXS data were collected on Instrument B21 in several sessions between 2019 to 2021 at the Diamond Light Source, Didcot, UK, in both batch and size-exclusion chromatography (SEC-SAXS) modes (36). B21 operated at a ring energy of 3 GeV, with a sample-to-detector distance of 4.014 m (Dectris). The detector has a resolution of 1475 × 1679 pixels (pixel size 172 × 172 mm), giving a *Q* range from 0.04 to 4 nm^-1^ (where *Q* = 4π sin *θ*/*λ*; 2*θ* is the scattering angle and *λ* is the wavelength). The intensity was calibrated in absolute units using a scattering reference buffer. Predialyzed samples of both native and deglycosylated SCR fragments and CFH were prepared in Hepes buffer, with concentrations ranging from 0.5 to 5.0 mg/ml and loaded onto a 96-well plate. For batch mode operations, an automatic sampler introduced 35 μl of sample from the plate into a temperature-controlled quartz cell capillary with a diameter of 1.5 mm. Data were captured as sets of 30 frames, each with an exposure time of 1 s, and acquired in duplicate to ensure reproducibility. After an automated inspection for minimum radiation damage, frames were subtracted from the reference buffer and averaged (37, 38). For the SEC-SAXS runs, 50 μl of the sample from the plate were injected onto a KW402.5 HPLC column (Shodex) connected to an Agilent HPLC system (Agilent). The software ScÅtter (version VI) was utilized for HPLC buffer subtraction, data reduction, and concentration normalization (36).

The reduced SAXS scattering curves *I(Q)* were analyzed using the SCT suite of programs (39) and ATSAS (Version 3.1.3) (40). This process yielded the radius of gyration (*R*_*G*_), cross-sectional radius of gyration (*R*_*XS*_), and maximum dimensions. The *R*_*G*_ value was derived from the Guinier analyses of the *I(Q)* curve at low *Q* ranges, representing the average solution structural elongation from the center of the protein, and the forward scattering at zero angle I(0) (41). For all the samples, the *Q.R*_*G*_ range up to 1.3 was used to yield satisfactory Guinier values:

$$\ln\;I\left(Q\right)=\ln\;I\left(0\right)-\;\frac{{R_{G}}^{2}Q^{2}}{3}$$

For an elongated protein such as CFH, the average radius of gyration of the cross-sectional structure (*R*_*XS*_) was calculated using a larger *Q* fit range than that used for the *R*_*G*_ values:

$$\ln\left[I\left(Q\right)Q\right]={\left[I\left(Q\right)Q\right]}_{Q\to 0}-\frac{{R_{XS}}^{2}Q^{2}}{2}$$

The scattering curve *I(Q)* was transformed from reciprocal space into real space using the program GNOM to give the distance distribution function *P(r)* (42, 43):

$$P\left(r\right)=\frac{1}{2\pi^{2}}\int_{0}^{\infty }I\left(Q\right)Qr\;\sin\left(Qr\right)dQ$$

The *P(r)* curve signifies the distribution of all interatomic distances, denoted by r, among all volume elements within the macromolecule. This *P(r)* curve gives *L*, representing the greatest dimension of a macromolecule, along with the *M1* peak, which correspond to the most frequently occurring distance vectors within the SCR structure and the *M1* and *M2* peaks for CFH.

### Atomistic modeling of the SCR fragments and CFH

CFH is comprised of 20 SCR domains with eight glycans (Fig. 1). Initially, the molecular structure of full-length CFH was taken from its most recent scattering modeling (6). This starting model originated from earlier NMR and crystal structures for 17 SCR domains and three SCR homology models for SCR-9, SCR-14, and SCR-17. This CFH model incorporated eight biantennary disialylated glycans, two of which are on SCR-17 and SCR-18. Previously, a library of 29,715 physically plausible CFH models suitable for SAXS curve fitting was created (6). The best-fit models from that earlier study were used to create SCR structures for the five fragments of this study in their monomeric state. Those for SCR-16/20 were unaltered. The SCR-16/18H, SCR-17/18H, SCR-18H, and SCR-17H structures were modified by incorporating the C-terminal sequence ALEQKLISEEDLNSAVDHHHHHH to these SCR models using MODELLER version 10.3 (44). Account was taken of the hyperglycosylated Man_8–18_GlcNAc_2_ glycan chains created by *Pichia* expression systems on SCR-17 and SCR-18 (Fig. 1*A*). The CHARMGUI GlycanReader tool was used to build and attach these glycans to Asn1011 and Asn1077 (45, 46, 47). The CHARMM-GUI software was utilized to generate the CHARMM force field and PSF inputs required for the NAMD energy minimizations and simulations in SASSIE-web (44, 45, 46, 47, 48, 49). Once the two glycans were added to SCR-17 and SCR-18 and approved by GlycanReader, bash scripts finalized the nomenclature and numbering of the glycan and protein atoms to align with the experimental protein. All fragments, both with and without glycans, were then energy minimized using NAMD version 2.14 and the CHARMM36 force field over 50,000 steps (45, 46). The creation of dimeric SCR fragments for the SAXS curve comparisons was essentially arbitrary, given that the dimers were formed from glycan contacts and not protein contacts. For comparison purposes, attempts were made to assemble putative models for dimeric structures using the neural networking software AlphaFold Multimer (50).

The SasCalc module in SASSIE-web simulated the theoretical scattering curve for each SCR or CFH model that was created (51, 52, 53). In order to compare the simulations with the experiments, the concentration-extrapolated experimental curves at low *Q* were interpolated to zero *Q* using MATLAB software. Following this, the *Q* spacing was evenly distributed among the data points. This procedure generated 761 *Q* values ranging from 0.0 to 1.0 nm^-1^ for the experimental SAXS curve. To evaluate the fit for each model against experimental SAXS data, a theoretical scattering curve was generated from each model. These curves were then compared with the experimental X-ray scattering curve, utilizing the R-factor function in SASSIE-web, which quantified the similarity between the theoretical and experimental curves. Lower R-factor values represent better fits:

$$R=\frac{\sum \left\|\left\|I_{Expt}\left(Qi\right)\right\|-\;\eta \;\left\|I_{Model}\left(Qi\right)\right\|\right\|}{\sum \left\|I_{Expt}\left(Qi\right)\right\|}\times 100$$

where Qi is the *Q* value of the ith data point; $I_{Expt}\left(Qi\right)$ is the experimental scattering intensity; and $I_{Model}\left(Qi\right)$ is the theoretical modeled scattering intensity (39). The libraries of SCR or CFH models produced an *R*-factor *versus R*_*G*_ distribution which is generally V-shaped. The best-fitting 100 models were determined from their R-factors. The model with the smallest R-factor was identified as the best-fit model. The wireframe renditions of these best models were created using SASSIE-web and PyMol software.

### SPR data collection and analyses for CFH

SPR experiments to determine dissociation constants *K*_*D*_ were carried out on a Biacore X100 instrument (Cytiva). In the binding assay, both streptavidin and a carboxymethylated dextran (CM5) sensor chips were used (Cytiva). To evaluate the binding kinetics between C3b and each of glycosylated and deglycosylated CFH, purified C3b was dialyzed against 10 mM NaCH_3_COO, pH 4.5, then amine-coupled to a CM5 sensor surface using the standard amine-coupling procedure (NHS/EDC coupling kit, Cytiva), according to the manufacturer's manual and Biacore X100 standard immobilization procedure. The C3b immobilization onto the CM5 sensor chip sample flow cell achieved 12,497 RU. High RU values ensured a robust and detectable signal during the interaction studies. Given the expected low affinity and transient nature of the interactions between CFH and C3b, higher immobilization levels were necessary to achieve an adequate signal-to-noise ratio. The 12,497 RU was within the limit of the CM5 chip and the X100 instrument. The reference flow cell was intentionally kept empty. Each assay was conducted at 25 °C using a Hepes running buffer, enhanced with 0.005% vol/vol surfactant Tween20. Each of glycosylated and deglycosylated CFH were introduced over the two flow cells in 5 to 8 concentrations, ranging from 0.05 μM to 7.75 μM. These concentrations were achieved *via* sequential twofold dilutions and were injected at a rate of 20 μl/min using a single-cycle kinetics program. The running buffer was also injected using the same program for the purpose of background subtraction. Regeneration after each run was achieved by pulsing with 10 mM sodium acetate buffer, 5 M NaCl, 0.05 mM EDTA, pH 7.0, across both flow cells twice for 45 s.

To determine the self-association kinetics between glycosylated and deglycosylated CFH, an EZ-Link Biotinylation kit from Thermo Scientific was utilized (Thermo Fisher Scientific). Biotinylated glycosylated CFH was immobilized onto the sensor chip's sample flow cell, reaching 781 RU, while the reference flow cell was left empty. Each of glycosylated and deglycosylated CFH were then introduced over the two flow cells in 5 to 8 concentrations, this time spanning from 0.01 μM to 1.8 μM, prepared *via* sequential twofold dilutions. A flow rate of 20 μl/min was used again, using a single-cycle kinetics program, with the running buffer introduced in the same manner to subtract the background.

A 1:1 binding model was used through the Biacore X100 Evaluation Software 2.1 to determine the dissociation constants *K*_*D*_. The affinity fits were achieved by fitting the data points to a hyperbolic function, which utilized the Michaelis–Menten model in the Enzyme Kinetics module of Origin 2021 (OriginLab) where *p*^*2*^ signifies the *K*_*D*_ value, with X being the analyte concentration and Y being the response in RU (54):

$$Y=\frac{p^{1}X}{p^{2}+X}$$

## Data availability

All data are contained within this manuscript. The scattering models were deposited in the SASBDB database (https://www.sasbdb.org/) (55)) with the reference codes SASDUT2 (Pichia SCR-17/18 after PNGase F treatment) and SASDUU2 (produced in *E. coli*).

## Supporting information

This article contains supporting information.

## Conflict of interest

The authors declare that they have no conflicts of interest with the contents of this article.
