# Supplementary figures and images for: The SCR-17 and SCR-18 glycans in human complement factor H enhance its regulatory function
Source: J Biol Chem. 2024 Aug 2;300(9):107624. doi: 10.1016/j.jbc.2024.107624 (PMC11417181; doi:10.1016/j.jbc.2024.107624)

**Supplementary Material**

Figure S1: Full SDS gel photograph for Figure 2B of SCR-16/20 and SCR-16/18H.

**
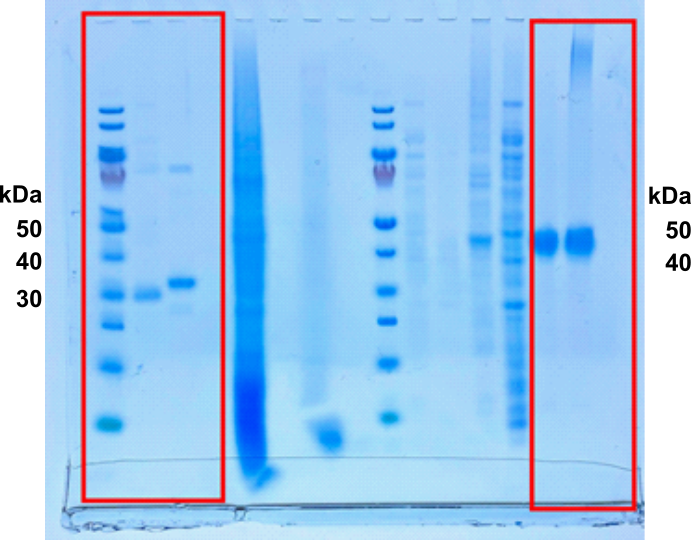
**

Supplement: Supplementary — Figure [file mmc1.docx]
